# Placental small extracellular vesicles from normal pregnancy and gestational diabetes increase insulin gene transcription and content in β cells

**DOI:** 10.1042/CS20241782

**Published:** 2024-11-20

**Authors:** Faheem Seedat, Neva Kandzija, Michael J. Ellis, Shuhan Jiang, Asselzhan Sarbalina, James Bancroft, Edward Drydale, Svenja S. Hester, Roman Fischer, Alisha N. Wade, M. Irina Stefana, John A. Todd, Manu Vatish

**Affiliations:** 1 Centre for Human Genetics, Nuffield Department of Medicine, University of Oxford, Oxford OX3 7BN, U.K.; 2 Nuffield Department of Women's and Reproductive Health, University of Oxford, Oxford OX3 9DU, U.K.; 3 Target Discovery Institute, Centre for Medicines Discovery, Nuffield Department of Medicine, University of Oxford, Oxford OX3 7FZ, U.K.; 4 Research in Metabolism and Endocrinology, Department of Internal Medicine, School of Clinical Medicine, Faculty of Health Sciences, University of the Witwatersrand, Johannesburg 2193, South Africa; 5 Division of Endocrinology, Diabetes and Metabolism, Perelman School of Medicine, University of Pennsylvania, 3400 Civic Center Boulevard, Philadelphia, PA 19104, U.S.A.

**Keywords:** beta cell, extracellular vesicles, gestational diabetes, insulin, placenta, pregnancy

## Abstract

Insulin secretion increases progressively during pregnancy to maintain normal maternal blood glucose levels. The placenta plays a crucial role in this process by releasing hormones and extracellular vesicles into the maternal circulation, which drive significant changes in pregnancy physiology. Placental extracellular vesicles, which are detectable in the plasma of pregnant women, have been shown to signal peripheral tissues and contribute to pregnancy-related conditions. While studies using murine models have demonstrated that extracellular vesicles can modulate insulin secretion in pancreatic islets, it remains unclear whether these effects translate to human biology. Understanding how placental signals enhance insulin synthesis and secretion from β cells could be pivotal in developing new therapies for diabetes. In our study, we isolated placental small extracellular vesicles from human placentae and utilised the human β cell line, EndoC-βH3, to investigate their effects on β-cell function *in vitro*. Our results indicate that human β cells internalise placental small extracellular vesicles, leading to enhanced insulin gene expression and increased insulin content within the β cells. Moreover, these vesicles up-regulated the expression of Annexin A1, a protein known to increase insulin content. This up-regulation of Annexin A1 holds promise as a potential mechanism by which placental small extracellular vesicles enhance insulin biosynthesis.

## Introduction

A multitude of physiological changes occur during pregnancy to support foetal growth and prepare the mother for childbirth [1]. Glucose is the principal energy source for the growing foetus and glucose homeostasis also undergoes changes during pregnancy [2]. Maternal insulin resistance increases approximately two-fold during pregnancy, which decreases glucose uptake by maternal tissues, allowing more glucose to be transferred across the placenta to the foetal circulation [2,3]. In parallel, maternal insulin secretion increases two to three-fold during pregnancy to maintain maternal and foetal euglycemia and aid foetal organogenesis [2–4]. Insulin is secreted by pancreatic β cells, found within the islets of Langerhans, along with a biologically inactive precursor, connecting peptide (C-peptide), which can be measured in serum as a surrogate of endogenous insulin production [5]. In type 1 diabetes mellitus, the destruction of β cells leads to the cessation of insulin production, and C-peptide levels become undetectable [5]. Interestingly, women with long-standing type 1 diabetes mellitus and undetectable C-peptide levels at conception exhibit an increase in C-peptide levels during pregnancy, indicating a recovery in β-cell function [6,7]. Pregnancy represents a state of enhanced β-cell function, and identifying the molecular mechanisms responsible for this change can help guide the development of pharmacotherapies for patients with diabetes.

In some women, hyperglycaemia develops during pregnancy. When glucose intolerance is first detected during pregnancy, it is classified as gestational diabetes mellitus (GDM) [8]. GDM is the most common medical complication of pregnancy and occurs in up to 14% of pregnant women [9]. GDM is associated with several short-term maternal and foetal complications such as increased rates of caesarean section, pre-eclampsia, large-for-gestational age infants, shoulder dystocia, and an increased rate of neonatal intensive care admission [9,10]. In the long term, women with GDM have a greater risk of type 2 diabetes and cardiometabolic disease, whilst their offspring are at a greater risk for metabolic disease and obesity [9]. Gaining insight into the molecular processes behind β-cell adaptations during pregnancy is essential for understanding GDM and developing therapies aimed at improving outcomes for both the mother and the child.

β-cell adaptations that enhance insulin secretion during pregnancy have been extensively studied in rodent models [11,12]. During pregnancy, β-cell mass, size, and proliferation all increase. Insulin gene (*INS*) transcription, insulin content, and insulin secretion also rise whereas the threshold for glucose-stimulated insulin secretion (GSIS) is reduced [11,12]. In humans, β cell mass is increased 1.4–2.4-fold during pregnancy [13,14]. However, there are few studies investigating whether the adaptations observed in mouse β cells during gestation also apply to human pregnancy.

Pregnancy associated β-cell adaptations are closely linked to the placenta [2]. Insulin secretion only begins to increase with the onset of placental development and rises proportionately with placental growth up to delivery [4]. After delivery, insulin secretion returns to its pre-pregnancy levels [2]. Several hormones secreted by the placenta, including prolactin, human placental lactogen, growth hormone, oestrogen, and progesterone, along with downstream transcriptional and proteomic changes, have been identified as potential regulators of β-cell function during pregnancy in rodent models [11,12,15].

In addition to hormones, the placenta also releases extracellular vesicles (EVs) into the maternal circulation [16]. EVs are lipid bilayer delimited particles released from cells and cannot self-replicate, as they lack a functional nucleus [17]. EVs can be classified based on their size — small EVs (sEVs) measure less than 200 nm, while medium/large EVs range from 200 to 1000 nm — or by their cell or tissue of origin, such as placental EVs, or by their biochemical composition, like CD63 or CD9 antigens [17]. EVs carry proteins, lipids, and genetic material and are able to communicate with peripheral tissues to influence downstream effects [16,18]. Placental small extracellular vesicles (psEVs) originate from the syncytiotrophoblast which forms the border of the placenta-maternal interface [16]. As the placenta grows with gestation, similar to the relationship between placental growth and insulin secretion, the release of psEVs from the placenta also corresponds to the rise in insulin secretion [19]. Of note, it is well described that psEVs are detected in the serum of pregnant women [19]. psEVs are distinct from sEVs originating from other tissue types and carry placental-specific proteins such as placental alkaline phosphatase (PLAP) and placental-specific genetic material, including the Chromosome 19 (C19) miRNA cluster encoding 59 mature miRNAs [19–21]. psEVs also act as signaling molecules, playing a role in physiological pregnancy adaptations and in pregnancy-related diseases [16,19]. Hence, like placental hormones, psEVs may also influence β cells and contribute to the enhancements in β-cell function observed during pregnancy.

The effect of psEVs on maternal β cells has not been fully elucidated. In a study using rodent islets, insulin secretion increased when exposed to psEVs isolated from the plasma of women with healthy normal pregnancy [22]. This effect was not present when rodent islets were treated with psEVs from women with GDM [21]. Human and rodent islets differ significantly in composition, architecture, vascularisation, gene expression, and functional responses [23]. Additionally, rodent β cells exhibit notable differences in protein-coding and long non-coding RNA expression compared to human β cells [24]. Due to these differences, it remains unclear whether the observations in rodent β cell and islet models are applicable to human pregnancy. The use of rodent models is a limitation that extends to much of the work using β cells due to the lack of satisfactory human β cell model systems and the challenges in acquiring human islets for scientific study. However, this limitation has been overcome by the development of a human β cell line, EndoC-βH3, originally derived from human foetal pancreatic tissue [25].

In the present study, we utilise the EndoC-βH3 cell line. Additionally, we isolate normal pregnancy and GDM psEVs directly from human placentae using a well-established *ex vivo* modified dual-lobe placental perfusion technique [26]. We treat EndoC-βH3 cells with psEVs to understand their effect on insulin gene (*INS*) expression, insulin content, GSIS, along with any proteomic alterations that might influence these effects. Using this approach, we are able to investigate the effect of psEVs on β cells using a human-based model system. Our hypothesis is that psEVs from normal and GDM pregnancy alter the biology of pancreatic β cells, potentially playing a role in enhancing β-cell function during pregnancy.

## Materials and methods

### Study participants

Placentae were collected from women with a normal or GDM pregnancy who underwent elective caesarean section at term. Normal pregnancy was defined as a healthy singleton pregnancy in a woman with no chronic or pregnancy related disease. GDM was diagnosed, as per the National Institute of Clinical Excellence 2015 criteria for GDM, when a fasting plasma glucose level measured ≥ 5.6 mmol/L, or when either a 1-hour plasma glucose level measured ≥ 10 mmol/L or a 2-hour plasma glucose level measured ≥ 8.5 mmol/litre following a 75 g oral glucose tolerance test (OGTT) performed at 24–28 weeks [27]. GDM participants with either a concomitant chronic disease or second pregnancy-related disease were excluded. Participants were recruited on the day of elective caesarean section, and all were at or beyond 37 weeks of gestation. A datasheet was completed from their clinical records detailing their age and clinical information.

### Isolation of psEVs and red blood cell small extracellular vesicles (RBCsEVs)

Placentae from 8 women with GDM and 8 women with normal pregnancies (Table 1) were perfused using an *ex vivo* modified dual-lobe placenta perfusion technique to isolate psEVs [26]. In principle this system mimics *in utero* placental circulation. After a successful placental perfusion, the maternal perfusate that is collected contains the products shed by the placenta which, *in vivo*, would re-enter the maternal circulatory system.

**Table 1:** Characteristics of study participants

	Normal pregnancy (*n* = 8)	Gestational diabetes mellitus (*n* = 8)	*p*-value
Age (yrs)	33.5 (32.3–39)	29.5 (23.8–34.3)	0.09
Body mass index (kg/m^2^)	25.5 (23–31)	27.8 (25.1–32.6)	0.27
Gestational age at delivery (wks)	39.2 (39.1–39.6)	39.2 (38.1–39.4)	0.49
Maximum systolic blood pressure (mmHg)	126 (118–134)	131 (128–139)	0.24
Maximum diastolic blood pressure (mmHg)	80 (77–84)	82 (79–85)	0.67
Haemoglobin A1c (%)	–	5 (4.8–5.6)	N/A
Gestational diabetes mellitus treatment			
Diet no. (%)		2 (25)	N/A
Metformin no. (%)	N/A	4 (50)	
Metformin and insulin no. (%)		2 (25)	
Male foetus no. (%)	4 (50)	4 (50)	0.4
Foetal birthweight (g)	3507.5 (3255–4340)	3657.5 (3455–4357.5)	0.88

*Data is described using median/interquartile range (IQR). Comparison’s using unpaired, two-sided Mann-Whitney tests; or when described using number (no.) and percentage (%) are compared using two-sided Fisher’s exact tests.

**Number (no.), metformin (metf) and insulin (Ins).

Medium 199 containing L-glutamine and Earle’s salts (Sigma, cat. no. M4530), bovine serum albumin (BSA) (Sigma, cat. no. A3294), 500 units/L of sodium heparin with the addition of 0.8% dextran (Sigma, cat. no. D1662) for perfusion of the foetal facing placental surface was used for the placental perfusion. A placental lobe/cotyledon without calcification or haemorrhage was selected and the artery and vein on the foetal facing placental surface cannulated. Foetal media was infused through the foetal circuit at a rate of 9 rpm and the outflow to the foetal circuit cylinder maintained at 80–100 mL per 20 minutes for the duration of the perfusion. Maternal media, supplemented with oxygen, was infused at a rate of 30 rpm through the maternal circuit onto the maternal facing surface of the placenta. Maternal perfusate that collected within the reservoir was recirculated through the circuit. The perfusion was continued for 3 hours, after which the remaining maternal perfusate was collected for ultracentrifugation.

RBCsEVs, used as a control for non-placental sEVs, were isolated from peripheral blood of healthy pregnant women, matched for chronological and gestational age. To isolate RBCsEVs, blood was collected in sodium citrate vacutainers (BD diagnostics, cat. no. 363080), centrifuged, and the erythrocytes were incubated with calcium chloride (Sigma, cat. no. C1016) and calcium ionophore A23187 (Sigma, cat. no. C7522) to stimulate RBC-EV formation. The reaction was stopped with EDTA (Thermo Fischer Scientific, cat. no. 15575020).

Maternal perfusate and the RBC solution were first centrifuged (Beckman Coulter Avanti J-20XP centrifuge and Beckman Coulter Js-5.3 swing out rotor) twice at 1,500 *g* for 10 minutes at 4°C to remove cell debris. psEVs and RBCsEVs were then separated sequentially using ultracentrifugation (Beckman L80 ultracentrifuge and Sorvall TST28.39 swing out rotor). Maternal perfusate and the RBC solution were ultracentrifuged at 10,000 *g* for 35 minutes to pellet medium/large placental- and RBC-EVs (200–1000 µm). The supernatant was filtered through a 0.22 µm stericup filter (Merck Millipore, cat. no. SCGPU02RE) and spun at 150,000 *g* for 120 minutes at 4°C to pellet psEVs and RBCsEVs (50–200 µm). The pellet was resuspended in filtered phosphate buffered saline (fPBS), prepared by filtering PBS (Gibco, cat. no. 10010023) through a 0.1 µm Thermo Scientific™ Nalgene™ Rapid-Flow™ Sterile Disposable Filter Unit with PES, CN, SFCA or Nylon Membrane (Thermo Fisher Scientific cat. no. 565-0010). Protein concentration was measured using a Pierce™ BCA protein assay (Thermo Fisher Scientific, cat. no. 23227). Aliquots of normal pregnancy psEVs, GDM psEVs, RBCsEVs, and control samples were stored at −80°C.

Following a successful a placental perfusion and differential ultracentrifugation, the final preparation contained sEVs and the medium in which they are carried - the sEV carrier medium. To accurately control for the effects of the sEV carrier medium in experiments, the initial maternal perfusion media—excluding the placental perfusion step—was processed by differential ultracentrifugation and resuspended in fPBS, creating the sEV carrier control.

sEVs were characterised by immunoblotting, nanoparticle tracking analysis (NTA) and transmission electron microscopy (TEM).

### Immunoblotting

Cell lysates and placental lysates (40 µg) or sEVs (20 µg) were boiled at 90°C for 10 minutes in 4X Laemmli sample buffer with 10% β-mercaptoethanol (Sigma, cat. no. M6250). Samples were separated on 4–15% Mini-PROTEAN TGX gels (Bio-Rad, cat. no. 4568084, 10-well gels, and 4561086, 15-well gels) and transferred to polyvinylidene difluoride membranes (Merck Millipore, cat. no. IPFL00010) using a Mini Trans-BLOT Cell (Bio-Rad, cat. no. 1703930). Membranes were blocked with 5% ECL Prime blocking agent (Cytiva, cat. no. RPN418) for 1 hour, incubated with primary antibodies overnight at 4°C (Supplementary Table S1), washed with tris-buffered saline (Thermo Fischer Scientific, J60764.K2) with 0.1% Tween (Bio-Rad, cat. no. 1610781) (TBS-T), and then incubated with secondary antibodies for 1 hour at room temperature (Supplementary Table S1). Membranes were dried in 100% methanol (Sigma, cat. no. 34860), imaged using the Licor Odyssey CLx Infrared Imaging System (LI-COR Biosciences) and visualised using Image studio software V.2 (LI-COR Biosciences).

### Nanoparticle tracking analysis

NTA was employed to assess the size distribution of normal pregnancy psEVs, GDM psEVs, and RBCsEVs using a Nanosight NS500 instrument with a 405 nm laser and sCMOS camera (Malvern). Instrument performance was verified using 100 nm silica latex standards (Malvern, cat. no. NTA4088). Samples were diluted in fPBS to achieve a particle concentration between 2 × 10^8^ and 1 × 10^9^ particles/mL and introduced using an automated syringe pump. The camera level was set to 12, and settings were kept consistent across all samples. Five 60-second recordings were captured for each sample, requiring a minimum of 500 valid tracks for analysis. The data were analysed using NTA software version 2.3, Build 033 (Malvern) to generate size distribution profiles and mean size profiles for normal pregnancy and GDM psEVs, as well as for RBCsEVs.

### Transmission electron microscopy

The morphology of normal pregnancy psEVs, GDM psEVs, and RBCsEVs were characterised using TEM. Samples were diluted with fPBS to achieve a concentration of 0.1-0.3 µg/µL. A 10 µL aliquot of the sEV solution was applied to freshly glow-discharged carbon formvar 300 mesh copper grids for 2 minutes, blotted with filter paper, stained with 2% uranyl acetate for 10 seconds, blotted again, and air-dried. The sEVs on the grid were negatively stained to enhance contrast with the background. Imaging was performed using a FEI Tecnai 12 transmission electron microscope operating at 120 kV, equipped with a Gatan OneView CMOS camera.

### Cell culture and differentiation

EndoC-βH3 cells (Human Cell Design, France) were cultured according to the supplier’s instructions in Dulbecco’s Modified Eagle Medium (DMEM), low glucose, GlutaMAX Supplement, pyruvate media (Gibco, cat. no. 21885-108) containing 2% bovine serum albumin (BSA) (Roche, cat. no. 10775835001), 10 mM nicotinamide (Sigma, cat. no. N3376), 50 µM β-2 mercaptoethanol (Gibco, cat. no. 31350010), 5.5 µg/mL transferrin (Sigma, cat. no. T8158), 6.6 ng/mL sodium selenite (Sigma, cat. no. T8158), and 100 units/mL penicillin and 100 µg/mL streptomycin (Gibco, cat. no. 15070063). Cells were passaged every 7 days and cultured at 37°C in a humidified incubator supplying air and 5% CO_2_. Cells were differentiated by adding 4-hydroxitamoxifen (1 µl/10mL) to culture media for 21 days to excise the immortalising genes (SV40 Large T cell antigen (*SV40LT*) and human telomerase reverse transcriptase *(hTERT*)) by CRE-ERT2 [25]. Differentiated cells were used at a confluency of 90-95% for experiments.

### psEV and RBCsEV labelling with fluorescent PKH26 and SytoRNA select dyes

Normal pregnancy psEVs, GDM psEVs, and RBCsEVs were labelled using the PKH26 and SytoRNA Select dyes (Table S1). PKH26 was used to label the sEV membrane and SytoRNA Select to label the RNA cargo within sEVs.

Normal pregnancy psEVs, GDM psEVs, RBCsEVs, and the sEV carrier control were mixed with Diluent C and PKH26 dye. The reaction was quenched with 10% BSA and serum-free media. Samples were ultracentrifuged over a continuous 30% sucrose (Sigma, cat. no. S0389) cushion at 150,000 *g*, resuspended, and filtered through a qEV1/35 nm size exclusion column (Izon Science) to remove dye aggregates. The qEV1/35 nm column was pre-flushed twice with two column volumes of cold fPBS. The sample was then resuspended in 500 µL of fPBS and loaded onto the column. Subsequently, fPBS was added to the column, and the void volume (1.5 mL) was collected. This step was repeated. Following the third addition of fPBS, the purified sEVs were collected in the elution fraction (1.5 mL). Elution volumes were combined and re-concentrated using an Amicon 4 mL Ultra Centrifugal Filter 100 kDa column (Merck Millipore) and centrifuged at 4000 *g* until the sample volume measured less than 500 µL.

SytoRNA Select dye was used to stain sEV RNA. Normal pregnancy psEVs, GDM psEVs, and sEV carrier controls were incubated with 1 mM of SytoRNA Select dye, re-isolated using the qEV1/35 nm column (as detailed above), and re-concentrated using the Amicon column. Protein concentrations post-staining were measured using a Pierce™ BCA assay.

### Visualisation and quantification of EndoC-βH3 sEV internalisation

Following labelling, PKH26-stained psEVs and controls were analysed by NTA to determine if dye aggregates were present and subsequently added to EndoC-βH3 cells. Cells were also treated with unlabelled psEVs. After incubation, internalisation of pre-labelled normal pregnancy psEVs, GDM psEVs, and RBCsEVs by EndoC-βH3 cells and PLAP signal in unlabelled psEV-treated EndoC-βH3 cells were visualised using a Leica SP8 X-SMD FLIM confocal microscope.

### Cell treatments with labelled and unlabelled psEVs and RBCsEVs to demonstrate sEV internalisation by EndoC-βH3 cells

EndoC-βH3 cells were seeded in 8-well µ-slides (Ibidi) and treated for 6 hours with 40 µg/mL of labelled or unlabelled normal pregnancy psEVs, GDM psEVs, RBCsEVs, and respective sEV carrier controls. Slides were labelled by immunocytochemistry. Cells were fixed and permeabilised with 4% paraformaldehyde (Thermo Fisher Scientific, cat. no. 043368.9M). When fluorescent dyes were used for labelling EndoC-βH3 cells (wheat germ agglutinin [WGA] for the cell membrane and Hoechst 33342 for the nuclei, Supplementary Table S1), the cells were incubated with the fluorescent dyes for 10 minutes at room temperature, washed, and imaged. When EndoC-βH3 cells were labelled by antibody immunostaining, samples were first blocked with 10% goat serum (Sigma, cat. no. G6767) and 0.1% Tween, incubated with primary antibodies overnight at 4°C, and then incubated with secondary antibodies for 30 minutes at room temperature. Wells were washed and the cells imaged.

Cells were imaged to detect fluorescent signals representing the psEV and RBCsEV membranes (labelled with PKH26) as well as their RNA cargo (labelled with SytoRNA Select) within EndoC-βH3 cells. Additionally, imaging was used to detect the presence of the psEV-specific protein PLAP within treated cells.

Imaging was carried out on a Leica SP8 X-SMD FLIM confocal microscope using a 63X 1.4 numerical aperture (NA) objective and LASX acquisition software. Images (30 µm^2^) were acquired with laser excitation at 410 nm, 504 nm, and 558 nm, and detected using HyD and PMT detectors. Confocal settings were consistent for each comparative condition.

### Real-time quantitative PCR to detect the C19 microcluster in psEV-treated EndoC-βH3 cells

To further confirm the internalisation of psEVs by EndoC-βH3 cells, real-time quantitative PCR (RT-qPCR) was used to detect placental-specific miRNAs (C19 microcluster) in EndoC-βH3 cells following treatment with psEVs. EndoC-βH3 cells were seeded into six-well plates and treated with normal pregnancy psEVs (*n* = 3), RBCsEVs (*n* = 3), and the sEV carrier control (*n* = 3) at 40 µg/m for 8 hours. Cells were lysed with TRIzol Reagent (Life Technologies, cat. no. 15596026), and RNA was extracted and purified using the miRNeasy Micro Kit (Qiagen, cat. no. 217084). Ten nanograms of isolated RNA was transcribed using the TaqMan Advanced miRNA cDNA Synthesis Kit (Thermo Fisher Scientific, cat. no. A28007) and transcribed cDNA was diluted 1:10. TaqMan miRNA assays for the placental-specific C19 miRNA cluster, and U6 snRNA, as the endogenous control, were used for RT-qPCR (Supplementary Table S2). TaqMan Fast Advanced Master Mix (Thermo Fisher Scientific, cat. no. 4444557), specific primers (Supplementary Table S2), and nuclease-free water (Invitrogen, cat. no. AM3395) were combined, and the RT-qPCR performed using the QuantStudio 6 Flex RT-PCR system (Thermo Fisher Scientific) according to the manufacturer’s protocol. All RT-qPCRs were performed in triplicate.

### Live cell psEV uptake assay and image analysis to quantify psEV internalisation by EndoC-βH3 cells

Cells and nuclei were labelled using MemGlow™ 488 (20 µM) and SiR-DNA 647 (100 nM), respectively (See Supplementary Table S1). Verapamil (10 µM), an efflux pump inhibitor, was added to ensure intracellular dye retention [28]. PKH26 labelled normal pregnancy psEVs were then added at doses of 10, 20, and 40 µg/mL, respectively, to three wells, with the PKH26-stained sEV carrier control as the fourth condition. Live imaging of PKH26-labelled psEV internalisation was performed on an Olympus SpinSR SoRa spinning disc confocal microscope.

Before the assay, nine points per well (250 µm^2^ each) were marked. These points were visualised throughout the assay and Z-stacks (10.5 µm thick, 21 planes) acquired. The assay ran over 12 hours, generating 180 time points at 4-minute intervals, with nine Z-stacks captured per well per time point (nine replicate images for analysis). Imaging was performed on an Olympus SpinSR SoRa spinning disc confocal microscope with a 60X 1.3 NA silicone objective and the 50 µm pinhole disc of a Yokogawa CSU-W1 spinning disc unit. The system was equipped with a Hammamatsu ORCA Fusion BT camera and excitation lasers at 405 nm, 488 nm, 561 nm, and 640 nm. The system was controlled using the CellSense software package and was equipped with full CO2, humidity, and temperature control.

Images were analysed using Arivis Vision 4D (v 4.1.2) image analysis software after constructing an analysis pipeline. In brief, a Gaussian blur was applied to the cells and nuclei channels. The Blob Finder operation segmented nuclei. An intensity threshold was used to segment the cells and PKH26 psEV volume, respectively. Nuclei counts, total cell volume, and internalised psEV volume detected within the segmented total cell volume for each Z-stack was measured. Internalised psEV volume was normalised to total cell volume per time point, averaged and the mean values plotted. Non-linear regression determined the best fit for each dose curve and EC50 values were compared using an F-test.

### RT-qPCR for *INS*

Differentiated EndoC-βH3 cells were treated with 40 µg/mL of GDM psEVs, normal pregnancy psEVs, RBCsEVs and sEV carrier control (*n* = 6 per experiment) and a time course was conducted to evaluate *INS* expression in normal glucose (5.5 mM) media at 24, 30, 36, 48, and 72 hours. For the follow-up experiment, media were changed to either normal or high glucose (20 mM) media at 24 hours post-sEV treatment, and cells lysed at 30 and 36 hours. For extractions at the different time points, cells were lysed in TRIzol reagent (Life Technologies, cat. no. 15596026). RNA was extracted using the Direct-zol-96 RNA Kit (Zymo Research, cat. no. R2070), and cDNA was transcribed using the LunaScript® RT SuperMix Kit (New England Biolabs, cat. no. E3010). *INS* and *TBP* (reference gene) TaqMan gene expression assays (Supplementary Table S2) were used for RT-qPCR analysis as previously described. Fold changes of *INS* expression in cells treated with normal pregnancy and GDM psEVs were compared to those in cells treated with the controls (RBCsEVs and sEV carrier control).

### Insulin ELISA for insulin content

Differentiated EndoC-βH3 cells were seeded into 96-well plates at a density of 4 × 10^4^ cells per well and treated with 40 µg/mL of GDM psEVs, normal pregnancy psEVs, RBCsEVs, and the sEV carrier control (*n* = 6 per group) for 40 hours. Media was removed, and cell numbers measured using the CyQUANT Direct Proliferation Assay (Thermo Fisher Scientific, cat. no. C35011). Cells were then lysed in acid ethanol (0.18 M HCl in 96% ethanol vol/vol) and insulin content measured using the Mercodia insulin ELISA Kit (Mercodia, cat. no. 10-1113-01). Insulin content from each well was normalised to the cell count per well.

### Glucose-stimulated insulin secretion assay

Differentiated EndoC-βH3 cells were seeded into 96-well plates at a density of 4 × 10^4^ cells per well and treated with 40 µg/mL of normal pregnancy psEVs, RBCsEVs and sEV carrier control (*n* = 4 per group) for 40 hours. GSIS was performed 40 hours post-treatment.

GSIS controls, to assess the adequacy of EndoC-βH3 insulin secretion, were also prepared: dimethyl sulfoxide (DMSO) (200 mM) (Sigma, cat. no. D4540) was added to both 1 mM and 20 mM glucose media, tolbutamide (200 mM, an insulin secretagogue) (Sigma, cat. no. T 0891) was added to 1 mM glucose media, and diazoxide (200 mM, an inhibitor of insulin secretion) (Sigma, cat. no. D9035) was added to 20 mM glucose media. EndoC-βH3 cells, pre-treated with normal pregnancy psEVs (*n* = 4 biological replicates), RBCsEVs (*n* = 3), the sEV carrier control (*n* = 3), and untreated cells (for secretion control testing), were placed in low-glucose starvation media (2.8 mM) overnight. The following day, the media was changed to no-glucose media (0 mM), and the cells were incubated for 1 hour at 37°C. After this incubation step, either 1 mM or 20 mM glucose media was added to each experimental group, respectively, and incubated for an additional hour at 37°C to stimulate glucose secretion. Following GSIS, the supernatant was removed from each well and centrifuged at 500 *g* for 5 minutes at 4°C, from this plate the supernatant was once more removed and placed in a different plate. This step was performed to ensure that no residual cells remained in the supernatant, thereby representing only the secreted insulin. Insulin levels were then measured in this supernatant using the Mercodia insulin ELISA Kit. From the plate containing the cells, the remaining media was removed from the wells and the cells lysed using acid ethanol. Insulin content was measured using the Mercodia insulin ELISA Kit. Secreted insulin was normalised to the insulin content per well. To confirm differential insulin secretion at low and high glucose concentrations, insulin secretion at 1 mM glucose was compared to 20 mM glucose.

### psEV cell treatments and protein extractions

Differentiated EndoC-βH3 cells were treated with 40 µg/mL of GDM psEVs, normal pregnancy psEVs, RBCsEVs, and the sEV carrier control in two separate experiments, each using different biological replicates. In the first experiment, each condition (GDM psEVs, normal pregnancy psEVs, RBCsEVs, and the sEV carrier control) had *n* = 3 biological replicates, while in the second experiment, *n* = 4 biological replicates were used. Across both experiments, a total of *n* = 7 biological replicates were utilised.

Cells were seeded into six-centimetre culture dishes and treated with 40 µg/mL of GDM psEVs, normal pregnancy psEVs, RBCsEVs, and the sEV carrier control. After 24 hours, cells were lysed on ice for 15 minutes using 1X RIPA buffer (Thermo Fisher Scientific, cat. no. 89900) with protease (Roche, cat. no. 11873580001) and phosphatase (Roche, cat. no. 4906837001) inhibitors. Lysates were centrifuged at 20,000 *g* for 20 minutes at 4°C, and the supernatants collected. Protein concentrations were quantified using a Pierce™ BCA assay.

### Liquid chromatography-mass spectrometry (LC-MS/MS)

Extracted proteins from cells were analysed by LC-MS/MS, which was performed in collaboration with the Target Discovery Institute (TDI), University of Oxford.

Protein lysates from EndoC-βH3 cells treated with GDM psEVs, normal pregnancy psEVs, RBCsEVs, and the sEV carrier control were prepared using FASP (Filter-Aided Sample Preparation) digestion as follows: the FASP filter (Vicacon® 500, Sartorius, VN01H02, 10kDA) was washed with 200 µL of 0.1% trifluoroacetic acid (TFA) (Sigma, cat. no. 80457) in 50% acetonitrile (ACN) (Sigma, cat. no. AX0156), followed by centrifugation at 14,300 rcf for 10 minutes. The sample was then loaded onto the filter and proteins were denatured with 200 µL of 8 M urea (Sigma, cat. no. U4883) (prepared by dissolving 4.8 g of urea in 10 mL of 100 mM triethylammonium bicarbonate [TEAB][Sigma, cat. no. T7408]) for 30 minutes at room temperature. For reduction, tris(2-carboxyethyl) phosphine (TCEP) (Sigma, cat. no. C7406) was added to a final concentration of 10 mM (by adding 4 µL of 0.5M TCEP) and incubated for 30 minutes at room temperature. Alkylation was performed by adding chloroacetamide (C-AA) (Sigma, cat. no. 22790) to a final concentration of 50 mM (20.4 µL of 0.5M C-AA) for 30 minutes in the dark. The liquid was then spun through at 14,300 rcf for 10 minutes. The filter was washed twice with 200 µL of 50 mM TEAB, each wash was followed by centrifugation at 14,300 rcf for 10 minutes to prepare for trypsin digestion. Prior to protease addition, the sample was transferred to a fresh tube. Digestion was carried out using 2 µg of trypsin in 200 µL of 50 mM TEAB, incubated overnight at 37°C. After digestion, the liquid was spun through and the flow-through was retained. The filter was washed with 200 µL of 0.1% TFA, and the flow-through was combined with the initial flow-through. The filter was then washed with 200 µL of 50% ACN in 0.1% TFA, and the flow-through was again combined. The combined flow-through was dried down using a speedvac vacuum concentrator and re-suspended in 50 µL of 5% formic acid (FA) (Sigma, cat. no. 5.33002) and 5% DMSO.

Sample loaded evotips were run using the high throughput Evosep One LC system connected to the TimsTOF Pro mass spectrometer (Bruker Daltonics). Peptides were analysed using the pre-built 100 samples / per day method (EvosepOne) with an 11.5 min gradient (total cycle time of 14.4 min) at a 1.2 µL/min flow rate. Tryptic peptides were transferred from the pre-loaded C18 evotips with a pre-build gradient to a sample loop and separated on a 150 µm × 8 cm C18 analytical column (Evosep Pepsep, 3 µm beads, 100 µm ID) with an overall gradient from 3 to 40% acetonitrile.

Mass spectrometry data were acquired in data-independent acquisition mode with parallel accumulation, serial fragmentation (diaPASEF) (oTOF control v6.0.0.12). The ion mobility window was set to 1/k0 start = 0.85 Vs/cm^2^ to 1/k0 end = 1.3 Vs/cm^2^, ramp time 100 ms with locked duty cycle, mass range 100–1700 m/z. MS/MS were acquired in 4 Parallel Accumulation Serial Fragmentation (PASEF) frames (3 cycles overlap). Target intensity was set to 6000 and threshold intensity 200.

The raw mass spectrometry (MS) data were analysed using a library-free approach with Data-Independent Acquisition by Neural Networks (DIA-NN, version 1.8.1). The analysis utilised the UniProt reference proteome for Homo sapiens (UPR_Homo sapiens_9606_UP000005640_20221003.fasta). To ensure high-confidence identifications, the false discovery rate (FDR) was maintained at 0.01. The Match Between Runs (MBR) feature was activated to enhance quantification accuracy across different datasets. For consistent and precise quantification, cross-run normalisation was executed using maximal label-free quantification (MaxLFQ).

### LC-MS/MS data analysis

LC-MS/MS data was analysed using the computational proteomics analysis software, Perseus (v2.0.11) [29]. All measured data were log2 transformed, biological replicates belonging to each treatment group were combined together as the control groups (sEV carrier controls and RBCsEVs), normal pregnancy psEVs and GDM psEVs. Rows were filtered for valid values necessitating at least 3 values per group and imputation applied following a normal distribution. Groups were compared by a Student’s *t*-test using permutation-based testing to correct for multiple comparisons. An FDR = 0.05 and S0 = 0.1 were applied to determine statistical significance. The principal component analysis (PCA) was performed using the in-built PCA function. Volcano plots were obtained by plotting (log10, Student’s *t*-test) against the fold change of the normalised mean MS intensities (log2).

### Statistical analysis

Prism 10 (2024) was used to perform statistical comparisons. Statistical tests were selected based on the characteristics of the acquired data and the comparisons required. For two-group comparisons, normally distributed data were analysed using the unpaired Student’s *t*-test, while non-parametric data were assessed with the Mann-Whitney test. For comparisons involving more than two groups, parametric data were analysed using a one-way ANOVA. Post-hoc tests used to compare individual groups included: Dunnett’s multiple comparisons test when comparing individual groups to the control group, Šídák’s multiple comparisons test for a small number of specific comparisons, and Tukey’s multiple comparisons test when all individual groups were compared to each other. For data that did not follow a normal distribution and involved more than two groups, the Kruskal-Wallis test was used, followed by Dunn’s multiple comparisons test for post-hoc analysis. Data are presented as mean ± standard error of the mean (SEM) or median ± interquartile range (IQR), as appropriate. Statistical significance was defined as *p* < 0.05. Statistical tests were selected based on the characteristics of the acquired data and the comparisons required.

## Results

### Isolated psEVs and RBCsEVs exhibited characteristic features of sEVs

**Figure 1:**
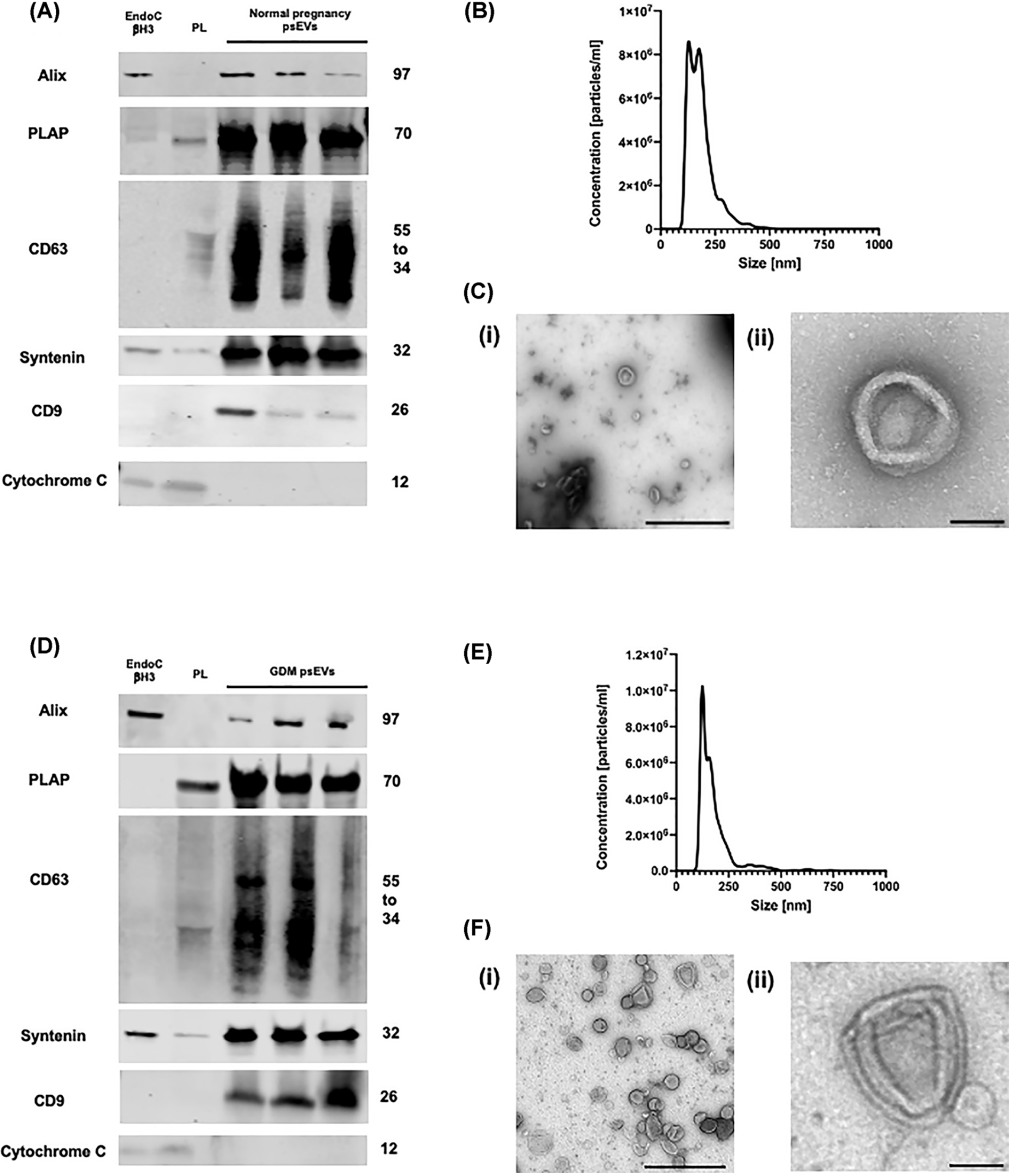
Characterisation of normal pregnancy and gestational diabetes mellitus (GDM) placental small extracellular vesicles (psEVs). A. and D. Immunoblots showing that A. normal pregnancy psEVs (n=3) and D. GDM psEVs (n=3) are enriched for extracellular vesicle markers and placental alkaline phosphatase (PLAP). The observed molecular weight is indicated for each protein. B. and E. Nanoparticle tracking analysis showing that B. normal pregnancy psEVs and D. GDM psEVs are within the expected size range for small EVs. C. and F. Transmission electron microscopy of C. normal pregnancy psEVs and F. GDM psEVs demonstrating the typical cup-shaped appearance of an extracellular vesicle. (i) Scale bar = 1000 nm, (ii) Scale bar = 100 nm. *PL – placental lysate.​ental lysate..

To confirm the successful isolation of normal pregnancy psEVs, GDM psEVs and RBCsEVs, respectively, we verified their characteristic immunophenotypic features by immunoblotting, NTA, and TEM. Immunoblotting confirmed that isolated psEVs and RBCsEVs were enriched for traditional EV markers: tetraspanins (CD63 and CD9) and an endosomal sorting complex required for transport machinery protein (Alix). Syntenin was present in all sets of sEVs. The presence of PLAP in psEVs confirmed their placental origin, while CD235a enrichment in RBCsEVs verified their red blood cell origin (Figure 1A [normal pregnancy psEVs] and 1D [GDM psEVs] and Supplementary Figure S1A [RBCsEVs]). Placental lysate was used as a positive control for the detection of PLAP. Cytochrome c, the mitochondrial protein used as a marker to illustrate co-isolation of contaminants along with sEVs, was absent in sEVs confirming the purity of the sEV isolation. NTA analysis detected a homogenous population of particles in line with the expected size distribution of sEVs (<200 nm). The mean and modal sizes of the psEVs were 184.5 nm ± 0.3 and 155.3 nm ± 10.8 for normal pregnancy psEVs, and 180.7 nm ± 3.8 and 125.2 nm ± 2.1 for GDM psEVs, respectively. For RBCsEVs, the mean size was 166.4 nm ± 2, and the modal size was 120.7 nm ± 2.5. Particles consistent with the morphological appearance of sEVs were visualised by TEM (Figure 1B,C [normal pregnancy psEVs], Figure 1E,F [GDM psEVs] and Supplementary Figure S1B,C [RBCsEVs]).

### sEVs are internalised by EndoC-βH3 cells

We aimed to determine whether normal and GDM pregnancy psEVs, as well as RBCsEVs, were internalised by EndoC-βH3 cells by using confocal microscopy to visualise the signal from pre-labelled psEVs and RBCsEVs within the cells. Additionally, we sought to detect the placental psEV-specific protein, PLAP, in EndoC-βH3 cells treated with unlabelled psEVs. Lastly, we aimed to confirm the imaging results by detecting the presence of psEV-specific miRNAs (C19 microcluster) in psEV-treated EndoC-βH3 cells.

Following the labeling of psEVs with PKH26 dye, we first reviewed their size by NTA to ensure that psEV size had not increased and that no dye aggregates, that may mimic sEVs, were detected in the sEV carrier control. The size of PKH26 labelled psEVs remained in the small EV range and their mean size measured 171.5 nm ±1.2 and modal size, 143 nm ± 4.3. Moreover, no small EV sized particles were detected by NTA in the sEV carrier control sample labelled with PKH26 (insufficient number of valid tracks < 100).

**Figure 2:**
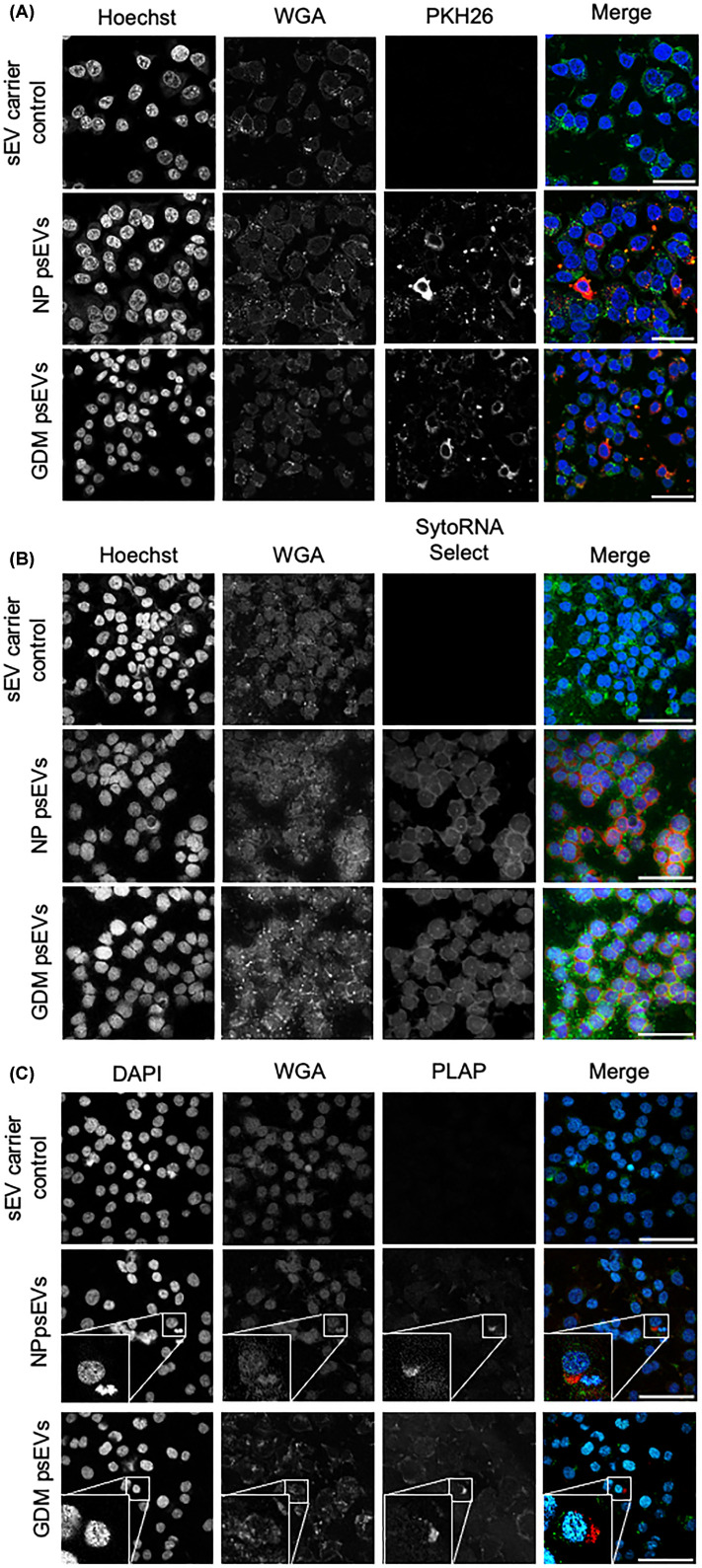
Normal pregnancy and gestational diabetes mellitus (GDM) placental small extracellular vesicle (psEV) internalisation by EndoC-βH3 cells visualised by confocal microscopy. A. Internalised PKH26-labelled psEVs (red) within EndoC-βH3 cells. Scale bar = 10 µm. B. Intracellular detection of SytoRNA Select dye (red) following treatment with SytoRNA Select-labelled psEVs. Scale bar = 20 µm. C. Immunocytochemistry showing placental alkaline phosphatase (PLAP) signal (red) within EndoC-βH3 cells following treatment with psEVs. An inset magnified image is show for each image in this panel. Scale bar = 20 µm. For all images, each channel is shown in grayscale, and the composite merged image is shown in colour (nuclei [DAPI] in blue, EndoC-βH3 cells in green and labelled psEVs [PKH26 and SytoRNA Select] or PLAP in red). *NP – normal pregnancy, sEV – small extracellular vesicle, WGA – wheat germ agglutinin.

**Figure 3:**
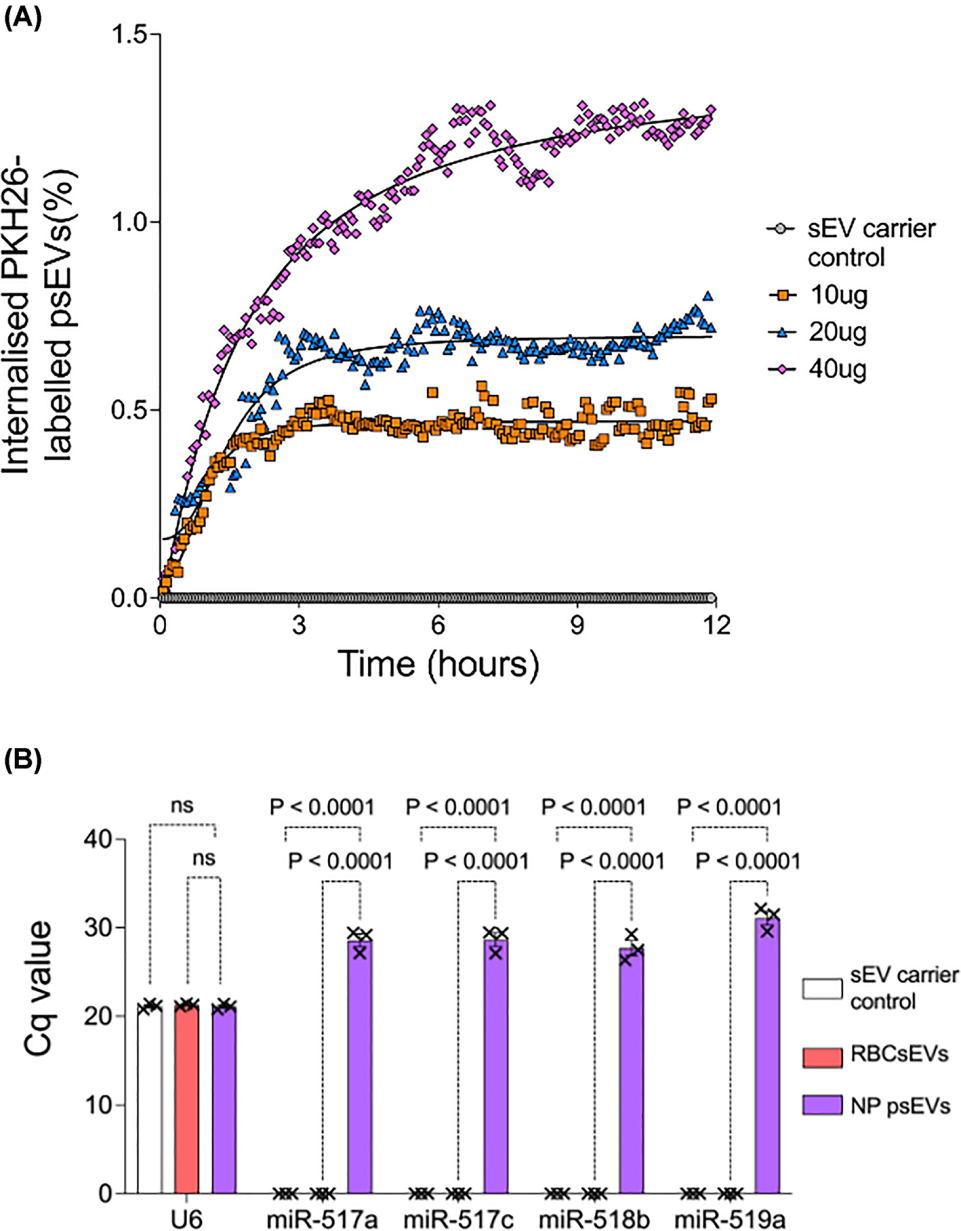
Time course of normal pregnancy placental small extracellular vesicle (psEV) internalisation in EndoC-βH3 cells and psEV internalisation confirmed by the presence of C19 psEV specific miRNA in cells treated with normal pregnancy psEVs. A. Time course of PKH26-labelled normal pregnancy psEV internalisation by EndoC-βH3 cells. The volume of internalised PKH26-labelled normal pregnancy psEVs within EndoC-βH3 cells is expressed as a percentage of total cell volume. Mean volume (n=9) measured at each time point for each dose is plotted. Non-linear regression was used to draw the line of best fit, showing a statistically significant difference in EC50 values (p=0.002). B. RT-qPCR demonstrating psEV internalisation. Placental-specific Chromosome 19 (C19) miRNA was only detected in EndoC-βH3 cells treated with normal pregnancy psEVs, not in those treated with red blood cell small extracellular vesicles (RBCsEVs) nor when treated with the small extracellular vesicle (sEV carrier control). U6 was used as the reference gene. The miRNAs analysed, belonging to the C19 microcluster, are shown on the x-axis. Differences were analysed by a one-way ANOVA for multiple comparisons. *p*-values are shown. ns = non-significant. *NP – normal pregnancy.​

EndoC-βH3 internalisation of PKH26 and SytoRNA Select labelled normal and GDM pregnancy psEVs and RBCsEVs was confirmed by confocal microscopy (Figure 2A,B [psEVs] and Supplementary Figure S2A and S2B [RBCsEVs]). Intracellular PLAP signal was detected in cells treated with unlabelled psEVs (Figure 2C). Details of the imaging pipeline used in the live-cell assay are shown in Supplementary Figure S3. The live-cell psEV uptake assay demonstrated that normal pregnancy psEVs were internalised in a dose- and time-dependent manner (Figure 3A). Maximal uptake occurred before 3 hours for the 10 µg dose, at 4-6 hours for the 20 µg dose, and uptake continued for up to 12 hours when a 40 µg dose was administered. Given that fixed cells treated with both normal pregnancy and GDM psEVs (Figure 2) showed no notable difference in psEV uptake patterns, and the similar size, morphology and immune profile shared by normal pregnancy and GDM psEVs we inferred that GDM psEV uptake followed the same dose- and time- dependent manner as that of normal pregnancy psEVs. Representative images illustrating the uptake of PKH26-labelled psEVs from the live-cell assay are shown in Supplementary Figure S4.

Further validation of psEV internalisation by EndoC-βH3 cells was achieved through RT-qPCR, which confirmed the presence of placental-specific C19 microcluster miRNA in cells treated with psEVs (*p* < 0.05) (Figure 3B).

### *INS* transcription and insulin content is increased by psEVs

To understand if psEVs conferred a direct effect on insulin biosynthesis we began by studying the effects of psEV treatments on *INS* transcription and insulin content.

Based on the live-cell psEV uptake assay we elected to use an sEV dose of 40 µg/mL and estimated that by 18 hours, all sEVs were likely internalised. We hypothesized that the earliest changes in *INS* expression would occur at 24 hours, six hours or more after maximal sEV uptake. We initially performed a time course to measure *INS* expression at several time intervals after the 24-hour timepoint to determine if any change in gene expression could be observed.

**Figure 4:**
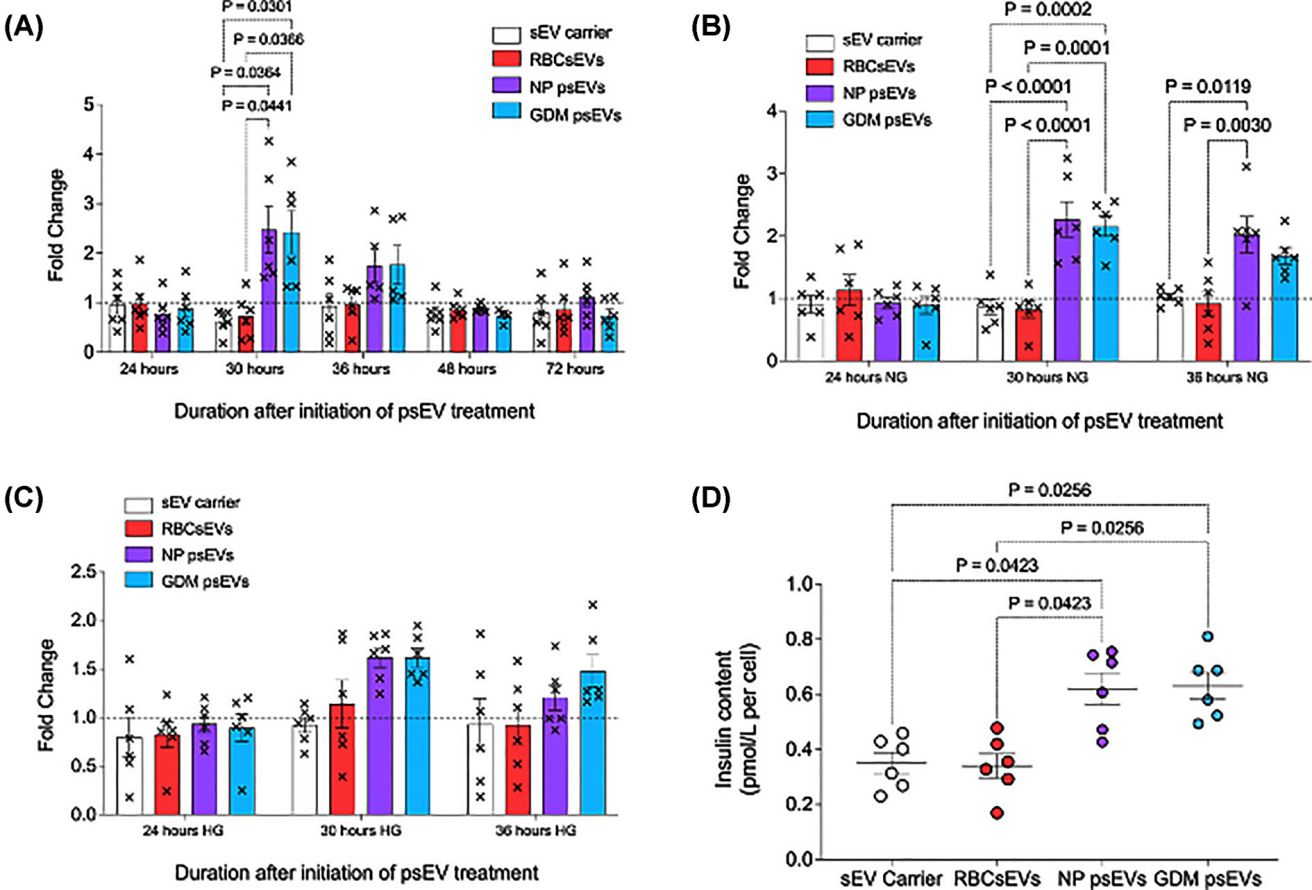
Normal pregnancy and gestational diabetes mellitus (GDM) placental small extracellular vesicle (psEVs) increase insulin gene (*INS*) transcription and insulin content in EndoC-βH3 cells. A., B., and C. *INS* transcription was measured in EndoC-βH3 cells treated with the small extracellular vesicle (sEV) carrier control, red blood cell small extracellular vesicles (RBCsEVs), and psEVs from normal pregnancy and GDM pregnancy. At each timepoint, the fold changes in *INS* expression for cells treated with psEVs from normal pregnancies (purple bar), GDM (blue bar), are compared to the controls (sEV carrier control [white bar]; RBCsEVs [red bar]). A. Time course of *INS* transcription in EndoC-βH3 cells (n=6 for each condition). *INS* expression is increased in normal pregnancy and GDM psEVs at 30 hours. B. INS transcription in EndoC-βH3 cells exposed to normal glucose (5.5 mM) and C. high glucose (20 mM) media (n=6 for each condition). *INS* expression is increased by normal pregnancy psEVs at 30 and 36 hours and by GDM psEVs at 30 hours in cells cultured in normal glucose media. No significant increases were detected in high glucose media. D. Insulin content is increased at 40 hours following treatment with both normal pregnancy (purple) and GDM (blue) psEVs compared to cells treated with sEV carrier controls (white) and RBCsEVs (red), n = 6 per condition. For A. B. C. differences were analysed using a one-way ANOVA followed by post-hoc testing using Dunnett’s multiple comparisons test for D. differences were analysed using a Kruskal-Wallis test, followed by Dunn’s multiple comparison for post-hoc testing. *p*-values for significant comparisons are shown. *HG – high glucose, NG – normal glucose, NP – normal pregnancy.

The time course showed *INS* transcription increased at 30 hours when compared to the controls in both normal pregnancy (fold change = 2.48 in normal pregnancy psEVs vs. fold change = 0.62 in sEV carrier, *p* = 0.0364 and fold change = 0.72 in RBCsEVs, *p* = 0.0441) and GDM (fold change = 2.42 in GDM psEVs vs. fold change in sEV carrier, *p* = 0.0301 and in RBCsEVs, *p* = 0.0366, respectively) psEV treatments. No change in *INS* expression was noted at 36, 48, and 72 hours (Figure 4A).

We then sought to determine if exposing cells to high glucose media (20 mM), to mimic hyperglycaemia, would have any effect on *INS* expression induced by psEVs. Following the psEV treatments, the increase in *INS* transcription was maintained in cells cultured in normal glucose media (5 mM) at 30 hours (fold change = 2.27 in normal pregnancy psEVs vs. fold change = 0.86 in sEV carrier, *p* < 0.0011 and fold change = 0.82 in RBCsEVs, *p* < 0.0001; fold change = 2.12 in GDM psEVs vs. fold changes in sEV carrier, *p* = 0.0002 and RBCsEVs, *p* = 0.0001, respectively) (Figure 4B) but was attenuated in cells cultured in high glucose media at 30 hours (fold change = 1.62 in normal pregnancy psEVs vs. fold change = 0.92 in sEV carrier, *p* = 0.1376 and fold change in RBCsEVs = 1.14, *p* = 0.6522; fold change = 1.62 in GDM psEVs vs. fold changes in sEV carrier, *p* = 0.1417 and RBCsEVs, *p* = 0.6610, respectively) (Figure 4C).

In a third replicate experiment performed using additional biological replicates (*n* = 8), *INS* transcription was once more increased at 30 hours in EndoC-βH3 cells cultured in normal glucose media when treated with normal pregnancy and GDM psEVs (fold change = 2.71 in normal pregnancy psEVs vs. fold change = 0.89 in sEV carrier, *p* < 0.0001 and fold change in RBCsEVs = 1.03, *p* < 0.0001; fold change = 2.19 in GDM psEVs vs. fold changes in sEV carrier, *p* < 0.0001 and RBCsEVs, *p* < 0.0001, respectively) (Supplementary Figure S5).

We then examined whether increases in *INS* transcription were translated to intracellular insulin content. Insulin content was increased in EndoC-βH3 cells treated with normal pregnancy psEVs (0.66 pmol/L, IQR: 0.46–0.75) and GDM psEVs (0.63 pmol/L, IQR: 0.52–0.73) relative to the sEV carrier controls (0.36 pmol/L, IQR: 0.26–0.44, *p* = 0.0423 for normal pregnancy psEVs and *p* = 0.0256 for GDM psEVs) and RBCsEVs (0.34 pmol/L, IQR: 0.26–0.43, *p* = 0.0423 for normal pregnancy psEVs and *p* = 0.0256 for GDM psEVs); however, no difference was noted between normal pregnancy and GDM psEVs (*p* > 0.99) (Figure 4D).

### psEVs have no effect on GSIS in EndoC-βH3 treated cells

We then studied whether increases in intracellular insulin content conferred by psEV treatments were also associated with changes in insulin secretion.

**Figure 5:**
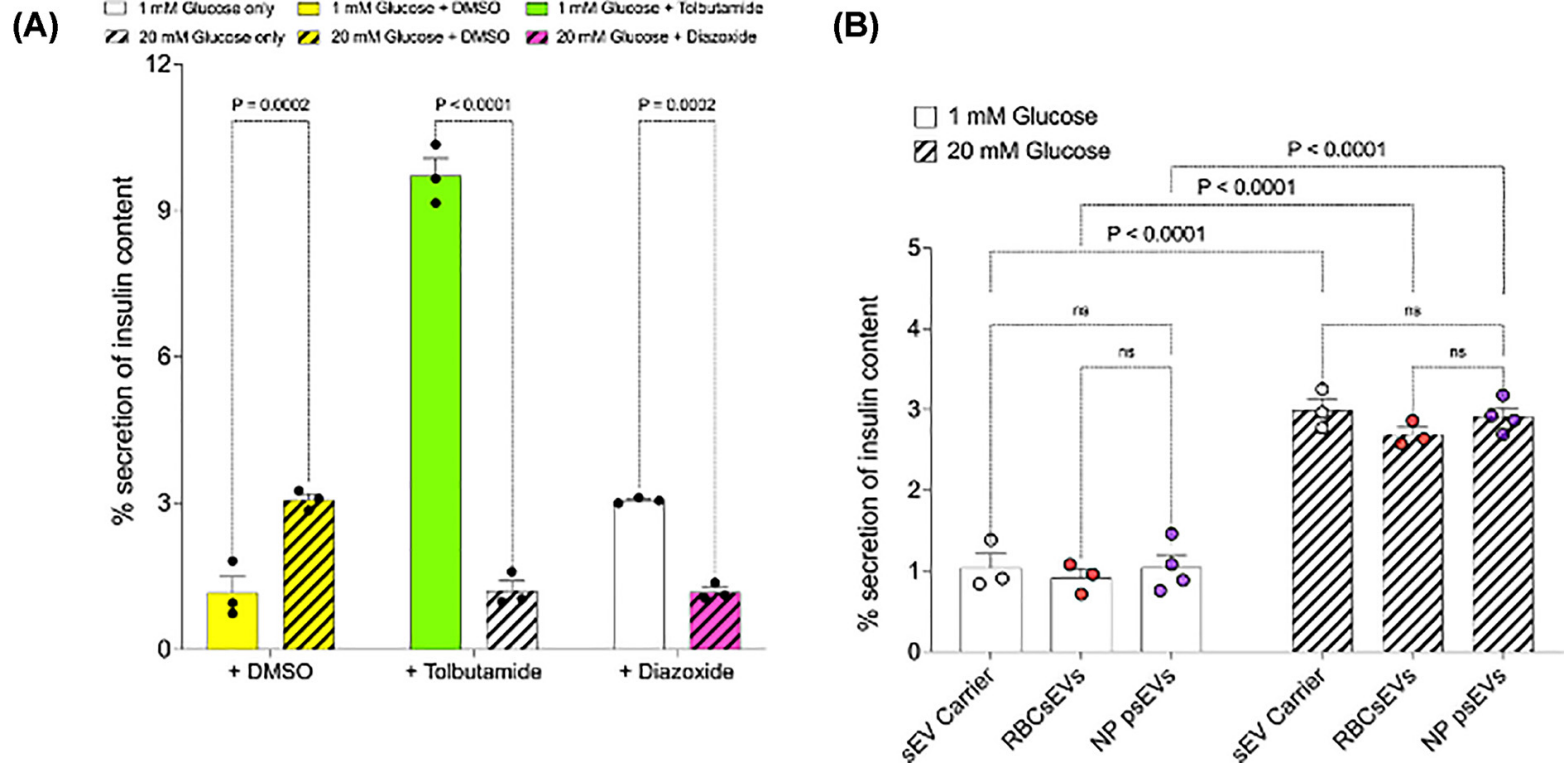
Normal pregnancy placental small extracellular vesicles (psEVs) have no effect on glucose-stimulated insulin secretion (GSIS) in EndoC-βH3 cells. A. GSIS assay showing insulin secretion by EndoC-βH3 cells cultured in basal media with 1 mM or 20 mM of glucose media containing 1mM and 20mM DMSO, and 1mM glucose media with tolbutamide or 20mM glucose media with diazoxide, respectively. Insulin secretion from cells adjusted correctly to varying glucose levels, staying low in media with 1 mM glucose and DMSO, but rising in media with 20 mM glucose in the presence of DMSO. Furthermore, tolbutamide, a known secretagogue, stimulated insulin release in 1 mM glucose media, while diazoxide, an inhibitor of insulin secretion, reduced insulin levels in 20 mM glucose media. Between-group differences were analysed using a one-way ANOVA, followed by Šídák's multiple comparisons test for individual group comparisons. Only * p*-values for significant comparisons are shown. B. GSIS assay of insulin secretion by EndoC-βH3 cells following treatment with the small extracellular vesicle (sEV) carrier (n=3), red blood cell small extracellular vesicles (RBCsEVs) (n=3) and normal pregnancy psEVs (n=4). An appropriate increase in insulin secretion was noted when EndoC-βH3 cells were exposed to 20 mM glucose compared to 1 mM glucose containing media for each condition. However, no differences in insulin secretion were detected when comparing normal pregnancy psEVs to controls in either 1 mM or 20 mM glucose containing media, respectively. Individual measures for sEV carrier controls (plotted in white), RBCsEVs (plotted in red), and normal pregnancy psEVs (plotted in purple) are shown. Differences were analysed using a one-way ANOVA, followed by Tukey’s multiple comparisons test for individual group comparisons. *NP – normal pregnancy.

EndoC-βH3 cells responded appropriately to GSIS controls, including: 1 mM and 20 mM glucose media containing DMSO, as well as to the pharmaceutical drugs added to cell culture media to stimulate or inhibit insulin secretion. Insulin secretion increased in the presence of 20 mM glucose media with DMSO relative to 1 mM glucose media with DMSO (20 mM glucose media with DMSO, 3.066% ± 0.116 vs. 1 mM glucose media with DMSO, 1.166% ± 0.329, * p* < 0.001). Insulin secretion was stimulated by the secretagogue tolbutamide in 1 mM glucose media (tobutamide in 1 mM glucose media, 9.722% ± 0.348 vs. 1 mM glucose media only 1.203% ± 0.196, *p* < 0.001) and inhibited by diazoxide, an inhibitor of insulin secretion, in 20 mM glucose media (diazoxide in 20 mM glucose media, 1.177% ± 0.099 vs. 20 mM glucose media only 3.052% ± 0.034, *p* < 0.001) (Figure 5A).

GSIS performed using EndoC-βH3 cells treated with the sEV carrier control, RBCsEVs, and normal pregnancy psEVs showed an appropriate reduction in insulin secretion in 1 mM glucose media and an increase in insulin secretion in 20 mM glucose media (sEV carrier in 1 mM glucose media, 1,046% ± 0.171 vs. sEV carrier in 20 mM glucose media, 2.844% ± 0.071, *p* < 0.0001; RBCsEVs in 1 mM glucose media, 0.092% ± 0.108 vs. RBCsEV in 20 mM glucose media, 2.693% ± 0.093, *p* < 0.0001; normal pregnancy psEVs in 1 mM glucose media, 1.049% ± 00.153 vs. normal pregnancy psEVs in 20 mM glucose media, 2.693% ± 0.093, *p* < 0.0001). However, when cells treated with controls (sEV carrier control and RBCsEVSs) were compared to those treated with normal pregnancy psEVs at the 1mM and 20mM glucose media concentrations, respectively, no difference in insulin secretion was observed in either low (1 mM) (normal pregnancy psEVs compared to sEV carrier, *p* = 0.9107; normal pregnancy psEVs compared to RBCsEVs,* p* = 0.4156) or high (20 mM) glucose media concentrations (normal pregnancy psEVs compared to sEV carrier, *p* = 0.6451; normal pregnancy psEVs compared to RBCsEVs, *p* = 0.2273) (Figure 5B).

### Expression of Annexin A1 is up-regulated by psEVs

To identify possible mechanisms contributing to the upregulation of *INS* expression and insulin content conferred by psEVs, we conducted an LC-MS/MS experiment to evaluate the proteome of EndoC-βH3 cells following treatment with normal pregnancy psEVs, GDM psEVs, RBCsEVs, and the sEV carrier control (*n* = 3 per group). We were interested in understanding whether psEVs alter the proteome of EndoC-βH3 cells and if any changes in protein abundance that were identified could mechanistically contribute to the observed increases in *INS* transcription and insulin content. Given that *INS* transcription increased at 30–36 hours post-psEV treatment, for any protein to affect *INS* transcription its’ abundance would need to be altered at an earlier time point to when we observed changes in *INS* expression. Hence, we chose to evaluate the proteome of psEV-treated EndoC-βH3 cells at 24 hours following psEV treatments which was 6–12 hours prior to the upregulation of *INS* transcription.

**Figure 6:**
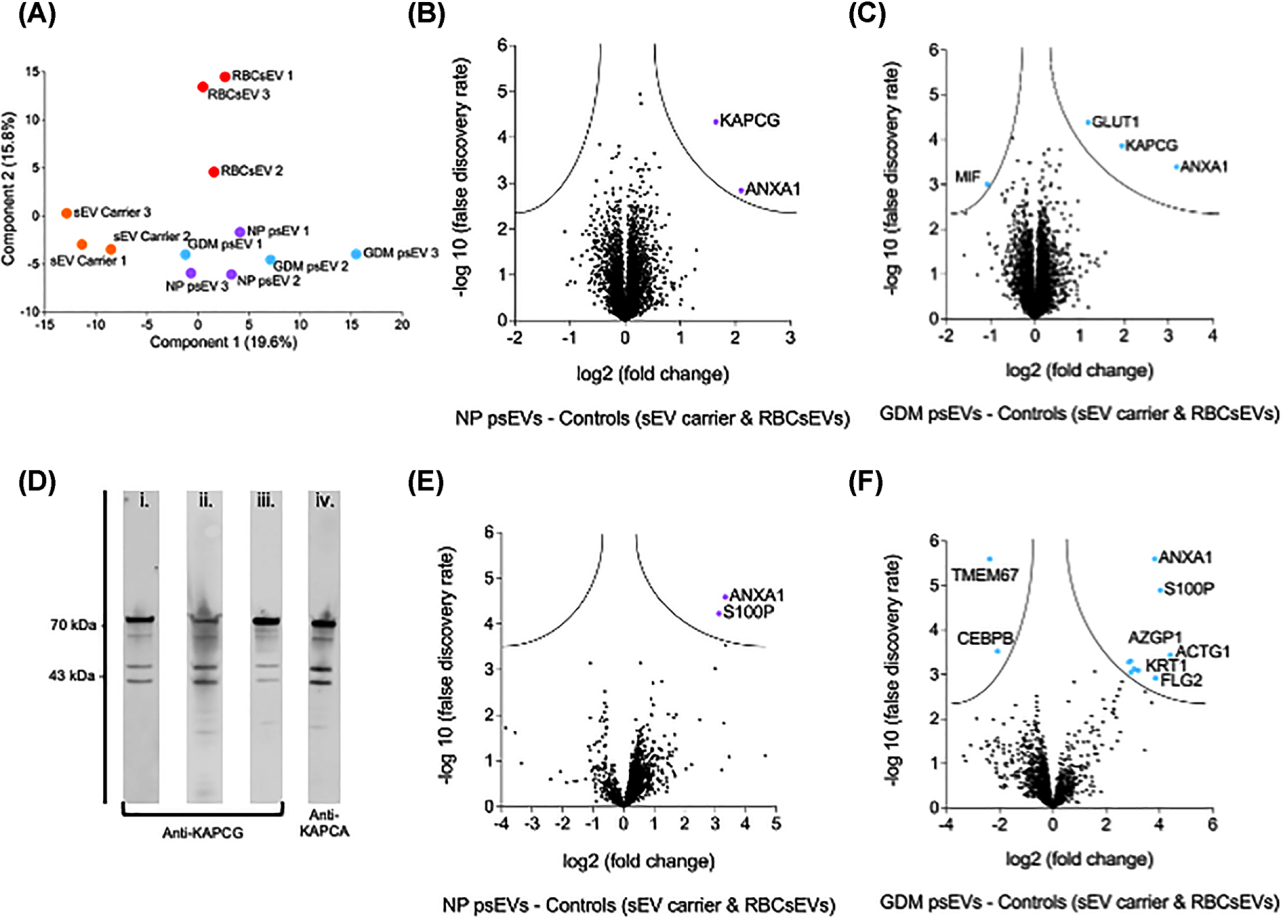
Annexin A1 (ANXA1) abundance is upregulated by treatment of in EndoC-βH3 cells with normal pregnancy and gestational diabetes mellitus (GDM) placental small extracellular vesicles (psEVs). A. Principal component analysis (liquid chromatography-mass spectrometry [LC-MS/MS] experiment 1) demonstrating clustering of different treatment groups based on their proteomic profiles. Each colour-coded cluster represents the proteomic data from one of the respective treatment groups: small extracellular vesicle (sEV) carrier control (orange), red blood cell small extracellular vesicles (RBCsEVs) (red), NP psEVs (purple), and GDM psEVs (blue). B and C. Volcano plots illustrating the differentially expressed proteins identified in the first LC-MS/MS experiment. The data compare protein abundance levels in EndoC-βH3 cells treated with (B) NP psEVs and (C) GDM psEVs against cells treated with controls (sEV carrier control and RBCsEVs). Analysis was conducted using n=3 biological replicates for each group. Significance was determined using a false discovery rate (FDR) of less than 0.05 and an S0 value of 0.1. The vertical axis shows the statistical significance (−log10 P-value), while the horizontal axis represents the magnitude of change (log2 fold change) for each protein. Proteins that met both the FDR and fold change thresholds are highlighted, indicating those that are significantly upregulated or downregulated. D. Immunoblot of recombinant protein kinase cAMP-activated catalytic subunit alpha (KAPCA) protein (H00005566-P01) using anti-protein kinase cAMP-activated catalytic subunit gamma (KAPCG) antibodies: i. sc-514087 (Santa Cruz Biotechnology USA), ii. ab108385 (Abcam UK), iii. abx301838 (Abbexa UK), iv. Anti-KAPCG antibody (67491-1-IG, Proteintech USA). A band at the predicted molecular weight of KAPCA + GST tag (70 kDa) is seen for all antibodies used, demonstrating non-specific binding by anti-KAPCG antibodies to the KAPCA recombinant protein. Molecular weight markers in kDa is shown as a ladder on the left. E and F. Volcano plots illustrating the differentially expressed proteins identified in the second LC-MS/MS experiment, which was conducted to replicate and validate the findings from the first experiment. The data compare protein abundance levels in EndoC-βH3 cells treated with (B) NP psEVs and (C) GDM psEVs against cells treated with controls (sEV carrier control and RBCsEVs). This experiment used a different set of biological replicates, with n=4 biological replicates for each group. Significance was determined using an FDR of less than 0.05, where FDR < 0.05 = -log 10 FDR > 1.3 and an S0 value of 0.1. The vertical axis displays statistical significance (−log10 FDR), while the horizontal axis represents the magnitude of change (log2 fold change) for each protein. Proteins that met both the FDR and fold change thresholds, indicating those that are significantly upregulated or downregulated, are highlighted. *NP – normal pregnancy.

The PCA plot is shown in Figure 6A. For the analysis of changes in protein abundance among the different treatment groups, proteins detected in cells treated with normal pregnancy psEVs and GDM psEVs were compared to those in cells treated with the sEV carrier control and RBCsEVs (control groups). In psEV treated cells, the abundance of Annexin A1 (ANXA1) (log2 fold change = 2.12, −log *p*-value = 2.8 [normal pregnancy psEVs] and 3.18, −log *p*-value = 3.4 [GDM psEVs]) and protein kinase cAMP-activated catalytic subunit gamma (KAPCG) (log2 fold change = 1.65, −log *p*-value = 4.3 [normal pregnancy psEVs] and 1.95, −log *p*-value = 3.9 [GDM psEVs]) were increased. Additionally, in GDM psEV treated cells, glucose transporter 1 (GLUT1) (log2 fold change = 1.2, −log *p*-value = 4.4) abundance was increased and macrophage migration inhibitory factor (MIF) abundance was reduced (log2 FC = −1.1, −log *p*-value = 3) (Figure 6B,C).

We aimed to validate the LC-MS/MS data in an independent replicate experiment using different biological samples by immunoblotting (*n* = 4 per group). Due to an established link between protein kinase A (PKA), *INS* expression, and insulin synthesis we first assessed KAPCG, the gamma catalytic subunit of PKA [30]. On assessing the amino acid sequence of KAPCG, it was found to share 80% homology with the protein kinase cAMP-activated catalytic subunit alpha (KAPCA) and beta subunits (KAPCB) [3,4]. Due to high sequence similarity between KAPCG and other PKA subunits (KAPCA and KAPCB) [30,31], antibodies with immunogens targeting regions with the least homology between PKA subunits were identified, purchased, and validated against recombinant full-length KAPCA protein (Abnova, cat. no. H00005566-P01) to determine their specificity. None of the three commercial antibodies tested were specific to KAPCG as they all detected the recombinant KAPCA protein. Hence, commercial antibodies (Supplementary Table S1) could not reliably detect KAPCG (Figure 6D). This made immunoblotting impractical, necessitating the repetition of the LC-MS/MS analysis to replicate and validate the original findings.

In the repeat replicate LC-MS/MS experiment, MIF expression was unchanged (log2 FC = −0.15, −log *p*-value = 0.27) in GDM psEV treated cells, GLUT1 expression was increased but was not significant (log2 FC – 1.45, −log *p*-value = 2.1) and KAPCG was not detected in any samples (Figure 6E,F). However, ANXA1 abundance was once more increased in EndoC-βH3 cells treated with normal pregnancy (log2 FC = 3.6, −log *p*-value = 4.3) and GDM psEVs (log2 FC = 3.82, −log *p*-value = 5.6) when compared to cells treated with the controls (sEV carrier control and RBCsEVs). This, replicated and validated the findings from the first LC-MS/MS experiment, eliminating the need to replicate the original findings by immunoblotting. Other differentially expressed proteins were also observed in this experiment (Figure 6E,F). However, these were not considered for further analysis, as their abundance was unchanged during the initial LC-MS/MS experiment and hence changes in their abundance had not been replicated across independent experiments.

## Discussion

We investigated the effect of psEVs on β-cell function using a human β cell line, EndoC-βH3. Our data demonstrate that psEVs are internalised by EndoC-βH3 cells and that psEVs from both normal and GDM pregnancy upregulate *INS* expression and increase β-cell insulin content. Additionally, we identified that ANXA1 is upregulated in EndoC-βH3 cells by psEVs which occurs at an earlier time point to the observed increases of *INS* and insulin content.

Our findings demonstrate the first example of psEV uptake by human β-cells, confirmed using confocal microscopy and by the intracellular detection of psEV-specific miRNAs. Both are established techniques for demonstrating sEV internalisation [21,32]. Limitations of lipophilic fluorescent dyes used to stain/label sEVs are reported in the literature; lipophilic dyes may self-aggregate to mimic sEVs and may cause a size shift following labelling [33–35]. To overcome these limitations we purified labelled sEVs using two steps and prepared appropriate dye labelling-controls using the sEV carrier control [34]. No nanosized particles mimicking dye aggregates were detected in the dye labelling-controls by NTA, while dye labelled-sEVs remained within the small EV range and no increase in size (size shift) was observed. Internalisation was also demonstrated using an alternative approach to sEV labelling by detecting psEV specific markers, PLAP protein and C19 miRNA cluster, within treated EndoC-βH3 cells. Live-cell imaging by confocal microscopy was used to directly visualise and quantify sEV internalisation by EndoC-βH3 cells. Our data on quantification of sEV internalisation aligns with previously published data using alternative techniques in other cell lines [21,36].

Insulin secretion and the number of circulating psEVs both increase with gestation, and a greater number of psEVs are present in the circulation of women with GDM compared to normal pregnancy [19]. Despite this association, few studies report on the effect of psEVs on β cells. In a recent study, psEVs were initially isolated from the plasma of pregnant women with normal and GDM pregnancy and then continuously infused into mice for four days [22]. Following the four-day infusion, pancreatic islets were isolated from each mouse and GSIS performed on the isolated islets. Insulin secretion was increased in mice infused with psEVs from women with normal pregnancy, however, no changes were observed in mice who received psEVs from women with GDM pregnancy [22]. In a second study, investigators isolated normal and GDM pregnancy psEVs from human placental explants and the transcriptomic profile of normal pregnancy and GDM psEVs were then compared. miR-320b was differentially expressed in psEVs isolated from the GDM psEVs [37]. Mouse whole islets and β cells were then transfected with miR-320b and GSIS performed, which demonstrated decreased insulin secretion, however, no changes in insulin synthesis or insulin content in islets were observed [37]. In contrast to these studies, we observed no increase in GSIS following treatments with psEVs. Nevertheless, our study is the first to demonstrate that both normal pregnancy and GDM psEVs increase *INS* transcription and insulin content in human β cells. No differences were observed between the effects conferred by psEVs isolated from placentae of women with normal versus GDM pregnancy. This suggests that the effect conferred by normal pregnancy and GDM psEVs on insulin biosynthetic pathways is analogous.

The lack of differences between normal and GDM pregnancy psEVs contrast with the observations in mice mentioned previously, where normal pregnancy psEVs increased GSIS, while GDM psEVs reduced GSIS [22]. Rodent models primarily examined insulin secretion rather than changes in insulin content. Changes in insulin synthesis and secretion may not directly correlate and may explain why we observed no difference in *INS* transcription or insulin content between cells treated with psEVs from normal and GDM pregnancy. Additionally, the differences in our findings compared to murine studies could also be attributed to the inherent biological distinctions that are present between human and mouse β cells [24]. Furthermore, previous rodent studies used whole islets rather than isolated β cells. It is possible that when using whole islets, β-cell function may have been influenced by the surrounding islet microenvironment, for example, by paracrine signalling from adjacent other islet cell types [38]. Finally, the observed differences may be explained by the varying methodologies used to isolate psEVs. In the murine studies discussed, psEVs were isolated from human plasma and human placental explants. We isolated psEVs using placental perfusion from a fresh placenta immediately following delivery, aiming to mimic the physiology of pregnancy. sEVs isolated from plasma samples may be contaminated with sEVs from other sources, lipoproteins, or other nano-sized particles, whilst EVs from placental explants, which are cultured over days, may be affected by the placental viability and culture conditions [39]. It is plausible that sEV co-isolates such as lipoproteins [39] or alterations in sEVs following placental explant culture may have contributed to the differences noted [40].

Beyond the differences between our study and previous studies conducted in mice, the lack of a difference observed between normal pregnancy and GDM psEVs may be due to additional factors. It is possible that, despite some distinctions between psEVs from normal pregnancy and GDM [41,42], the placental-specific cargo carried by psEVs that influences *INS* expression and insulin content, specifically, may be shared by both normal and GDM psEVs. Given the heterogeneity of GDM pathophysiology, where distinct subtypes (insulin resistance vs. insulin deficiency) have been identified [9], it is possible that psEVs released in GDM pregnancies may also reflect this heterogeneity. For instance, psEVs from insulin-deficient GDM patients could differ from those of insulin-resistant patients, and studying psEVs from the insulin-deficient population might reveal more pronounced effects on β-cell function. Another possibility is that β cells may possess compensatory mechanisms that mitigate the potentially harmful effects of GDM psEVs. These effects may only become evident when β cells are exposed to a high load of GDM psEVs, as might occur *in vivo* during GDM pregnancies [42], where the vesicle load is higher. A limitation of our study is that we did not assess psEV load and its impact on β-cell function, which should be further explored in future studies.

A further limitation of our study is that we did not perform GSIS on EndoC-βH3 cells treated with GDM psEVs. However, since we observed no differences in the effects on *INS* expression or insulin synthesis between normal pregnancy and GDM psEVs, we deemed it unlikely that GDM psEVs would affect GSIS as normal pregnancy psEVs had no effect on insulin secretion. Nevertheless, it is possible that GDM psEVs could exert distinct effects on GSIS, and exploring this interaction in future studies would be valuable. We found that *INS* expression was unchanged in cells cultured using high glucose media. This implies that elevated blood sugar levels negate the impact provided by psEVs on the insulin biosynthetic pathways in β cells cultured in normal glucose media. It is our hypothesis that glucotoxicity reduces psEV-induced *INS* gene upregulation and insulin synthesis, potentially explaining the β-cell dysfunction observed in patients with GDM. This reduction in β-cell function might explain why patients with GDM, who develop hyperglycaemia due to insulin resistance, develop compromised β-cell function despite the similar effects conferred by psEVs from women with normal pregnancy and GDM.

We observed that the ANXA1 abundance was increased in EndoC-βH3 cells by normal pregnancy and GDM psEVs. ANXA1, a 37kDa protein, belongs to the annexin superfamily of proteins, well-known for its anti-inflammatory effect [43]. It is found in circulation and localises to the cell membrane, cytoplasm, and nucleus [44]. Previous data has shown that ANXA1 is associated with β-cell function as well as glucose homeostasis and higher circulating levels are reported in type 2 diabetes-related obesity [45]. In a murine model, ANXA1 was identified as a key mediator of glucose homeostasis. ANXA1 knockout (ANXA1^−/−^) mice fed a high sugar, high fat diet had greater glucose intolerance compared to wild-type mice fed the same high sugar, high fat diet [46]. Notably, glucose levels declined, and OGTT improved when the aforementioned high sugar, high fat diet fed ANXA1^−/−^ mice were treated with human recombinant ANXA1 [46]. In rat islets, ANXA1 is colocalised with insulin-containing secretory vesicles, and high ANXA1 levels have also been detected within the cytosol and nucleus of β cells [47]. Glucose-induced phosphorylation at the serine residue of ANXA1 was associated with increased insulin secretion [47]. In MIN6N8a cells, a mouse pancreatic β-cell line, ANXA1 enhanced the second phase of insulin secretion by mobilising insulin vesicles and regulated insulin secretion through binding to a cell surface receptor [48,49]. Similar findings were reported in studies using rat islets [50,51]. Intracellular ANXA1, in rat islets, stimulate insulin secretion in an autocrine fashion [50]. Interestingly, with regards to insulin content, greater insulin content was measured in rat islets co-cultured with ANXA1 for 72 hours compared to controls [51]. In an overexpression model ANXA1 regulated the expression of *miR-26b* [52]. *miR-26* has been implicated in *INS* expression, as *miR-26* knockdown down-regulates insulin promoter activity and insulin mRNA synthesis [53]. We observed that psEVs increased ANXA1 expression in β cells followed by increases in *INS* expression and insulin content. Taken together, the existing literature on suggests ANXA1 may be a particularly interesting target in β cells. Clarifying the interaction between the psEV conferred upregulation of ANXA1 and increases in insulin synthesis warrants further investigation and may hold promise for the development of therapies to treat diabetes.

The strengths of the present study were the use of a human model system in the form of psEVs isolated from human placentae and the use of a human β cell line, EndoC-βH3. Moreover, the use of a placental perfusion-based system to isolate psEVs represents the most physiologically representative method to isolate psEVs and limits the contamination of sEVs from other sources. Although psEVs can be detected in the plasma of pregnant women, studying their effects on maternal pancreata *in vivo* is challenging due to ethical and practical restrictions associated with researching pregnant human subjects. Consequently, we adopted an *in vitro* experimental design. However, this approach has its limitations, and further verification of these findings is necessary, first in human islets and then through *in vivo* studies, ideally involving human subjects or ethically acceptable animal models that closely resemble human biology. The challenges posed by isolating psEVs using a placental perfusion system also limited the possible sample size included in the present study.

In conclusion, we have demonstrated that the human β-cell line, EndoC-βH3, internalises psEVs in a time- and dose-dependent manner. Once internalised, psEVs increase the expression of *INS* and insulin content in β-cells but have no effect on insulin secretion. Additionally, psEVs enhance the expression of ANXA1, which may serve as a potential mechanism to explain the observed effects of psEVs on insulin biosynthesis. Future work should aim to further elucidate the link between the increased abundance of ANXA1 induced by psEVs and their effect on upregulating insulin synthesis in β cells. Intracellular overexpression of ANXA1 should be employed to investigate its mechanistic role in upregulating insulin synthesis, specifically by investigating the molecular signalling pathways involved in this process. Additionally, these overexpression models can be applied to further explore ANXA1’s potential as a therapeutic target for diabetes, and to determine whether β cells exposed to glucotoxicity can maintain sufficient insulin production in the presence of elevated ANXA1 levels. We also aim to validate the findings of the present study through *in vivo* experiments.

## Clinical perspectives

- During pregnancy, insulin secretion increases to maintain maternal euglycemia, with the placenta playing a crucial role by signalling β cells through hormone release. Beyond hormones, the placenta secretes extracellular vesicles (psEVs) containing proteins and genetic material that can influence peripheral tissues. While rodent studies suggest psEVs can alter β cell function, their effects on human β cells remain unclear.
- Our study investigated the impact of psEVs on human β-cell function using the EndoC-βH3 cell line. We found that psEVs are internalised by these cells, leading to increased insulin gene (*INS*) expression and insulin content. Additionally, psEVs increased Annexin A1 (ANXA1) abundance, a protein potentially involved in enhancing insulin biosynthesis.
- These findings suggest that psEVs may regulate insulin synthesis during pregnancy, possibly through ANXA1 upregulation, contributing to improved β-cell function in pregnant women and offering insights for future therapeutic strategies in managing gestational diabetes mellitus (GDM).

## Supplementary Material

Supplementary Figures S1-S8 and Tables S1-S2

## Data Availability

The data included in the present study are available from the corresponding authors upon reasonable request.

## Open Access

Open access for this article was enabled by the participation of University of Oxford in an all-inclusive *Read & Publish* agreement with Portland Press and the Biochemical Society under a transformative agreement with JISC.

## Abbreviations

EV: extracellular vesicle
GSIS: glucose-stimulated insulin secretion
C-peptide: connecting peptide
NTA: nanoparticle tracking analysis
OGTT: oral glucose tolerance test
PLAP: placental alkaline phosphatase
fPBS: filtered phosphate buffered saline
TBS-T: Tris-buffered saline with 0.1% Tween
*SV40LT*: SV40 Large T cell antigen
*hTERT*: human telomerase reverse transcriptase
TEM: transmission electron microscopy
DMEM: Dulbecco’s Modified Eagle Medium
NA: numerical aperture
DMSO: dimethyl sulfoxide
TFA: trifluoroacetic acid
TEAB: triethylammonium bicarbonate
C-AA: chloroacetamide
FA: formic acid
GDM: gestational diabetes mellitus
psEV: placental small extracellular vesicle
sEV: small extracellular vesicle
RBCsEV: red blood cell small extracellular vesicle
SEM: standard error of the mean
IQR: interquartile range
FDR: false discovery rate
WGA: wheat germ agglutinin
RT-qPCR: real-time quantitative PCR
C19: chromosome 19
*INS*: insulin gene
NP: normal pregnancy
NG: normal glucose
HG: high glucose
LC-MS/MS: liquid chromatography-mass spectrometry
PCA: principle component analysis
ANXA1: Annexin A1
PKA: protein kinase A
KAPCG: protein kinase cAMP-activated catalytic subunit gamma
KAPCA: protein kinase cAMP-activated catalytic subunit alpha
KAPCB: protein kinase cAMP-activated catalytic subunit beta
GLUT1: glucose transporter 1
MIF: macrophage migration inhibitory factor
