# Supplementary material for: Placental small extracellular vesicles from normal pregnancy and gestational diabetes increase insulin gene transcription and content in β cells
Source: Clin Sci (Lond). 2024 Nov 20;138(22):1481–502. doi: 10.1042/CS20241782 (PMC11579211; doi:10.1042/CS20241782)
Supplement: Supplementary Figures S1-S8 and Tables S1-S2 [file CS-2024-1782_supp.pdf]

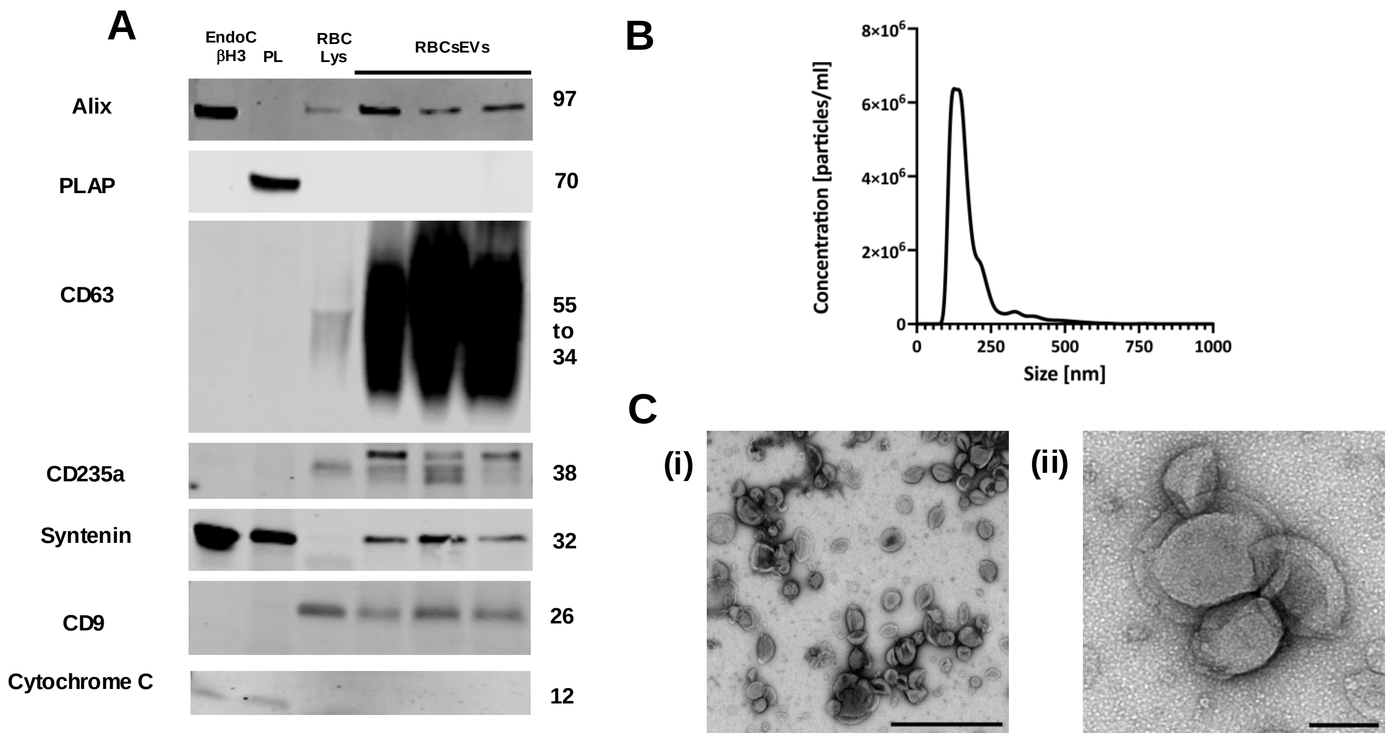

Supplementary Figure S1: Characterisation of red blood cell small extracellular vesicles (RBCsEVs).

A. Immunoblot showing RBCsEVs (n=3) enriched for EV markers and CD235a. Placental alkaline phosphatase (PLAP) was not detected in RBCsEVs. The observed molecular weight is indicated for each protein.

B. Nanoparticle tracking analysis of RBCsEVs showing RBCsEVs within the expected size range for small EVs.

C. Transmission electron microscopy of RBCsEVs demonstrating the typical EV cup-shaped appearance. (i) Scale bar = 1000 nm, (ii) Scale bar = 100 nm.

*Lys – lysate.

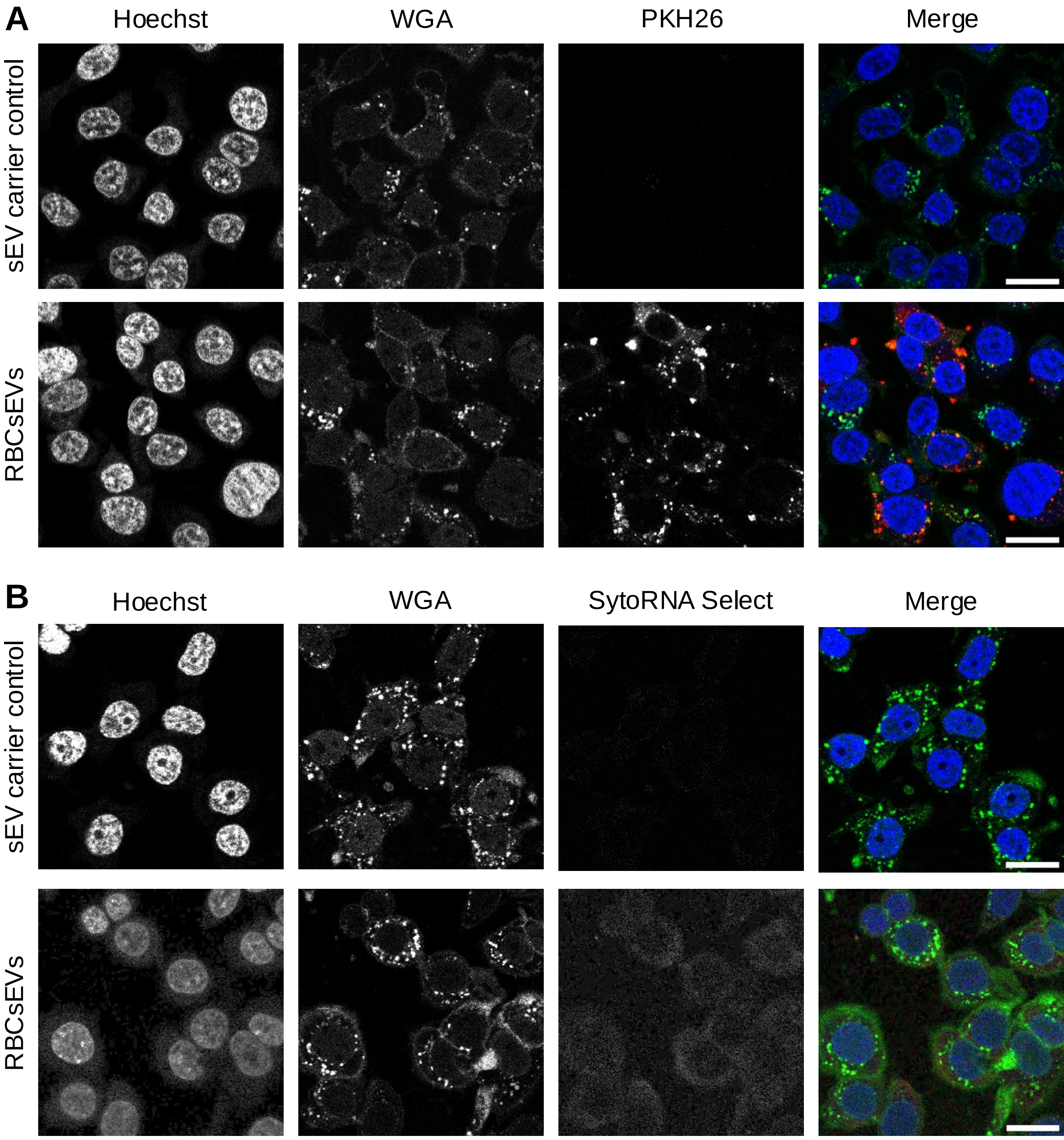

Supplementary Figure S2: Confocal microscopy demonstrating internalisation of red blood cell small extracellular vesicles (RBCsEVs) by EndoC-βH3 cells.

A. Internalised PKH26-labelled RBCsEVs (red) within EndoC-βH3 cells.
B. Intracellular detection of SytoRNA Select dye following treatment with SytoRNA Select-labelled RBCsEVs.

For all panels each channel is shown in grayscale and the composite merged iamge is shown in colour (nuclei [DAPI] in blue, EndoC-βH3 cells in green and labelled RBCsEVs in red). Scale bar = 10 µm.

*WGA – wheat germ agglutinin.

A. Single plane of acquired image

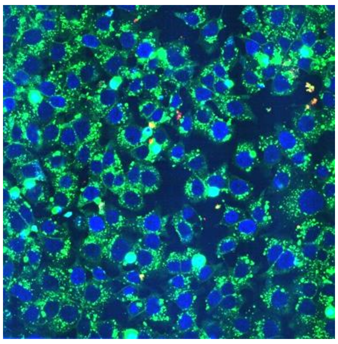

B. Segmented nuclei in red

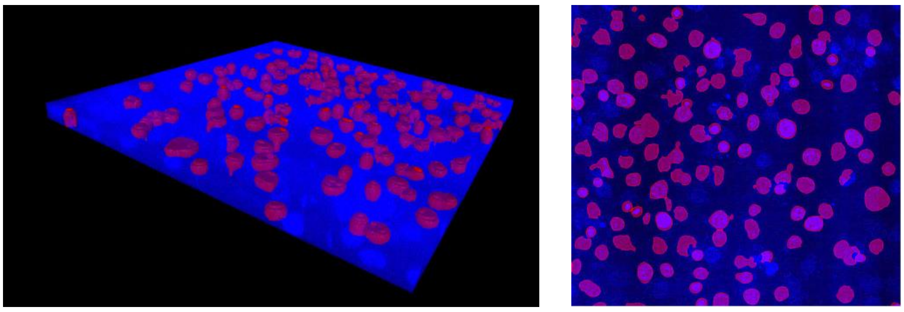

C. Segmented EndoC-βH3 cells in green

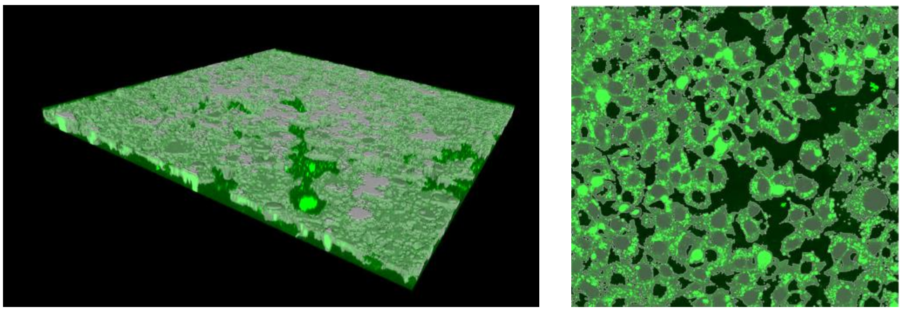

D. Segmented psEVs in cyan

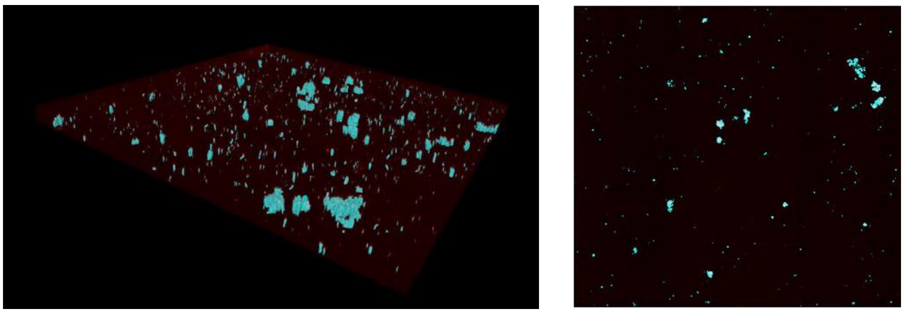

E. Internalised psEVs in pink, extracellular psEVs in yellow

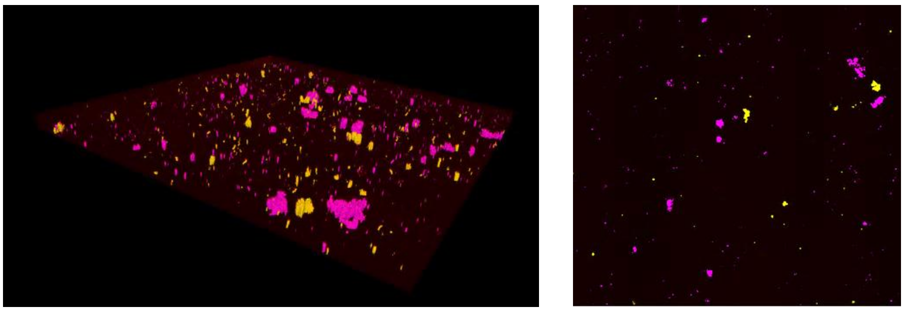

F. Composite image of all segmented structures

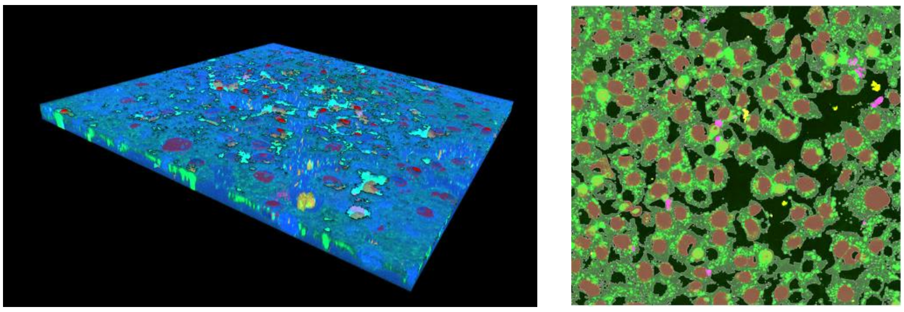

Supplementary Figure S3: Image analysis pipeline used to quantify normal pregnancy placental small extracellular vesicle (psEV) uptake by EndoC-βH3 cells using Arivis Vision 4D Image Analysis software.

Images at different stages of the analysis are shown to illustrate the methodological steps used to segment the cells, identify PKH26-labelled normal pregnancy psEVs, and quantify the volume of internalised psEVs within the cells. Left: 3D images; Right: 2D images. **A.** Single Z-position from converted image at timepoint 120 treated with 20 µg of PKH26-labelled psEVs. **B.** Nuclei (red) segmented using the blob finder tool. **C.** EndoC-βH3 cells segmented (green) using intensity thresholding. **D.** PKH26-labelled psEVs segmented using intensity thresholding. **E.** Internalised (pink) and extracellular (yellow) PKH26-labelled psEVs. **F.** Composite image showing segmentation of nuclei (red), cells (green), internalised psEVs (pink), and extracellular psEVs (yellow).

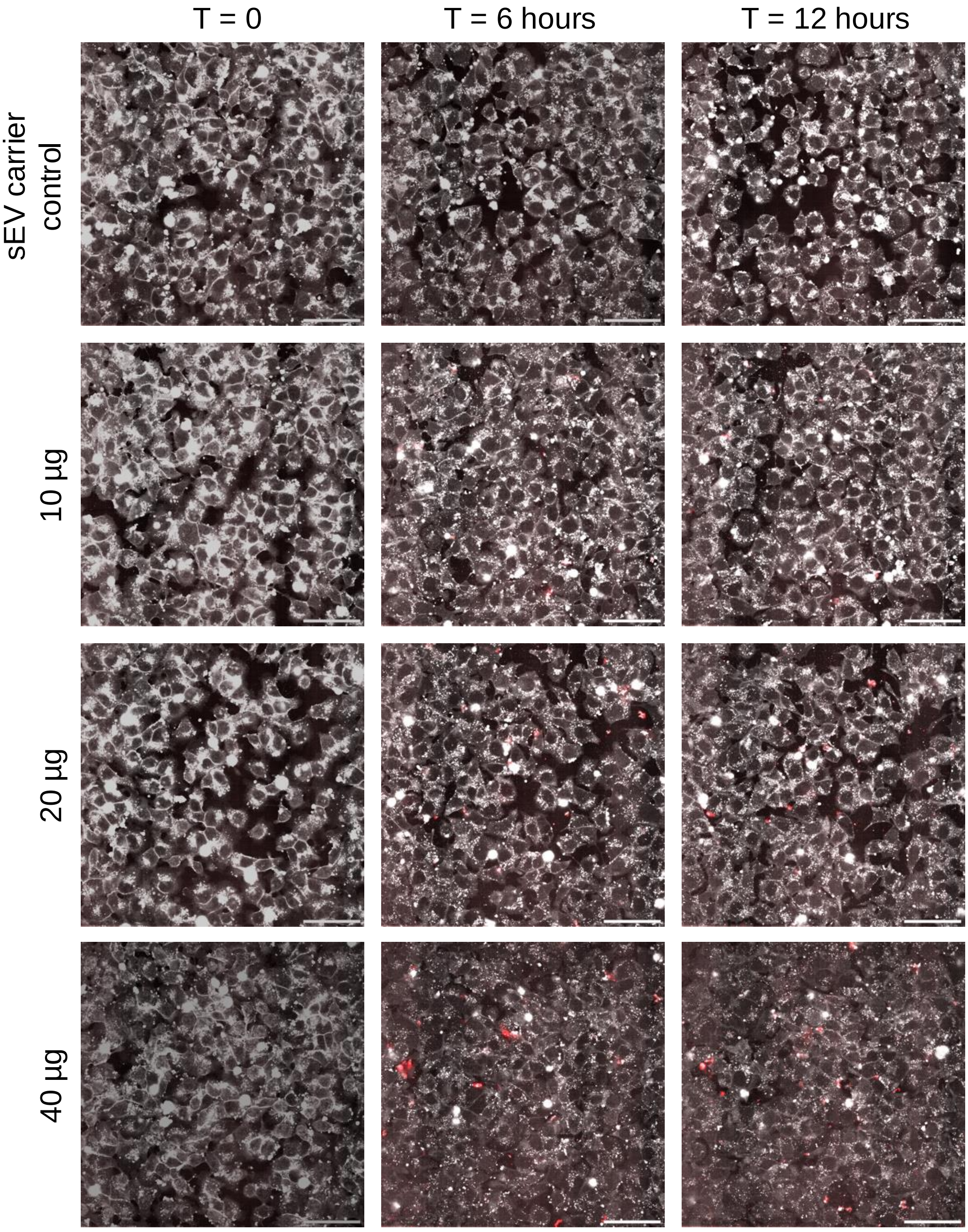

Supplementary Figure S4: Live-cell imaging of PKH26-labelled normal pregnancy placental small extracellular vesicle (psEV) internalisation at different timepoints.

Images at time points 0, 6, and 12 hours are shown for cells treated with the sEV carrier control and with the different doses of PKH26-labelled psEVs (10 µg, 20 µg, and 40 µg). Merged images are shown, which includes the nuclei and cells shown in grayscale and PKH26-labelled psEVs in red. Scale bar = 50 µm.

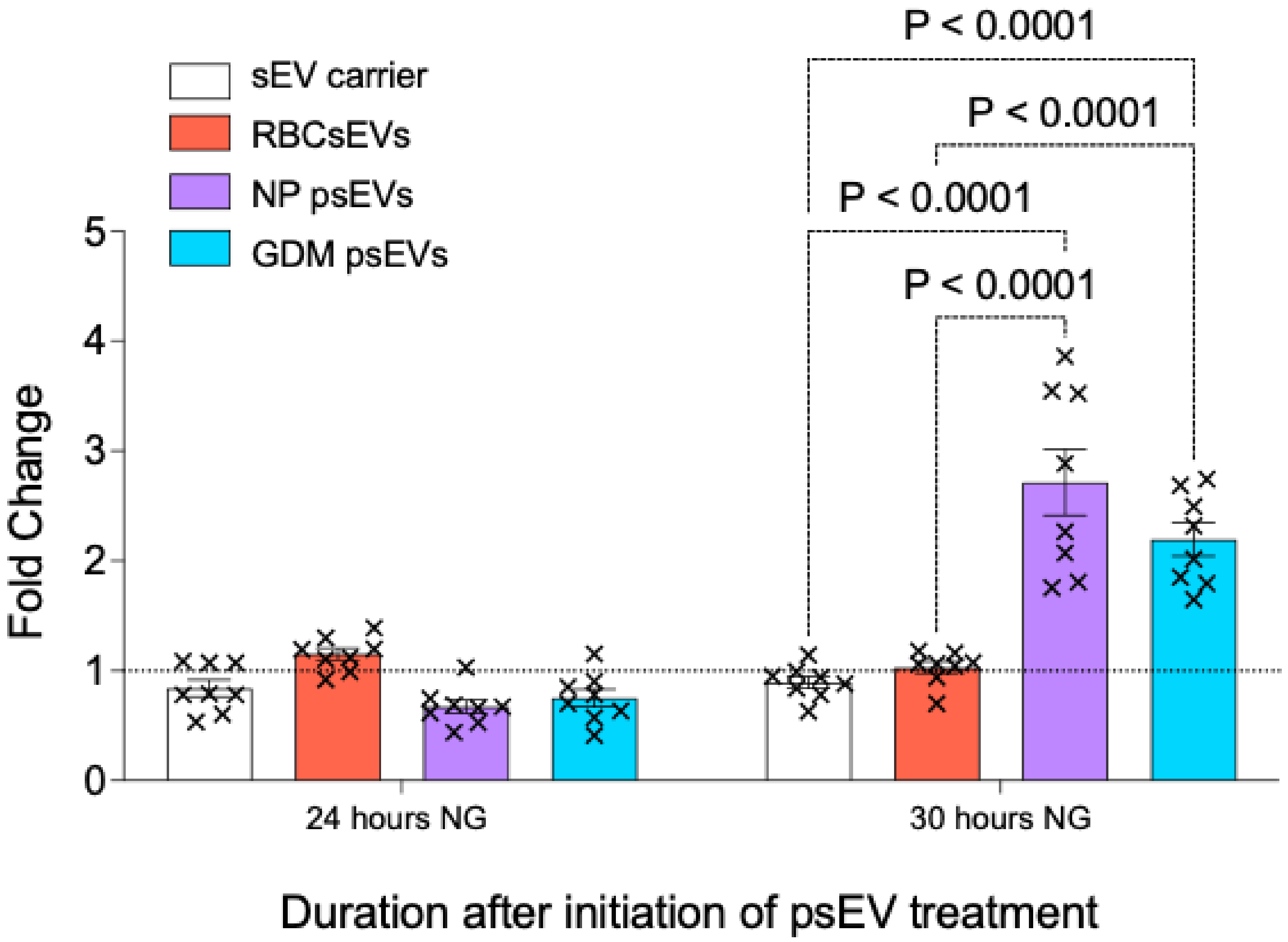

Supplementary Figure S5: Insulin gene (*INS)* transcription is consistently increased by normal pregnancy (purple bar) and gestational diabetes (GDM) (blue bar) placental small extracellular vesicles (psEVs) at 30 hours in EndoC-βH3 cells exposed to normal glucose media (n=8) relative to *INS* expression in the small extracellular vesicle (sEV) carrier (white bar) and red blood cell small extracellular vesicle (RBCsEVs) controls (red bar).

Differences were analysed using a one-way ANOVA followed by post-hoc testing using Dunnett's multiple comparisons test. p-values for significant comparisons are shown.

*NG – normal glucose, NP – normal pregnancy.

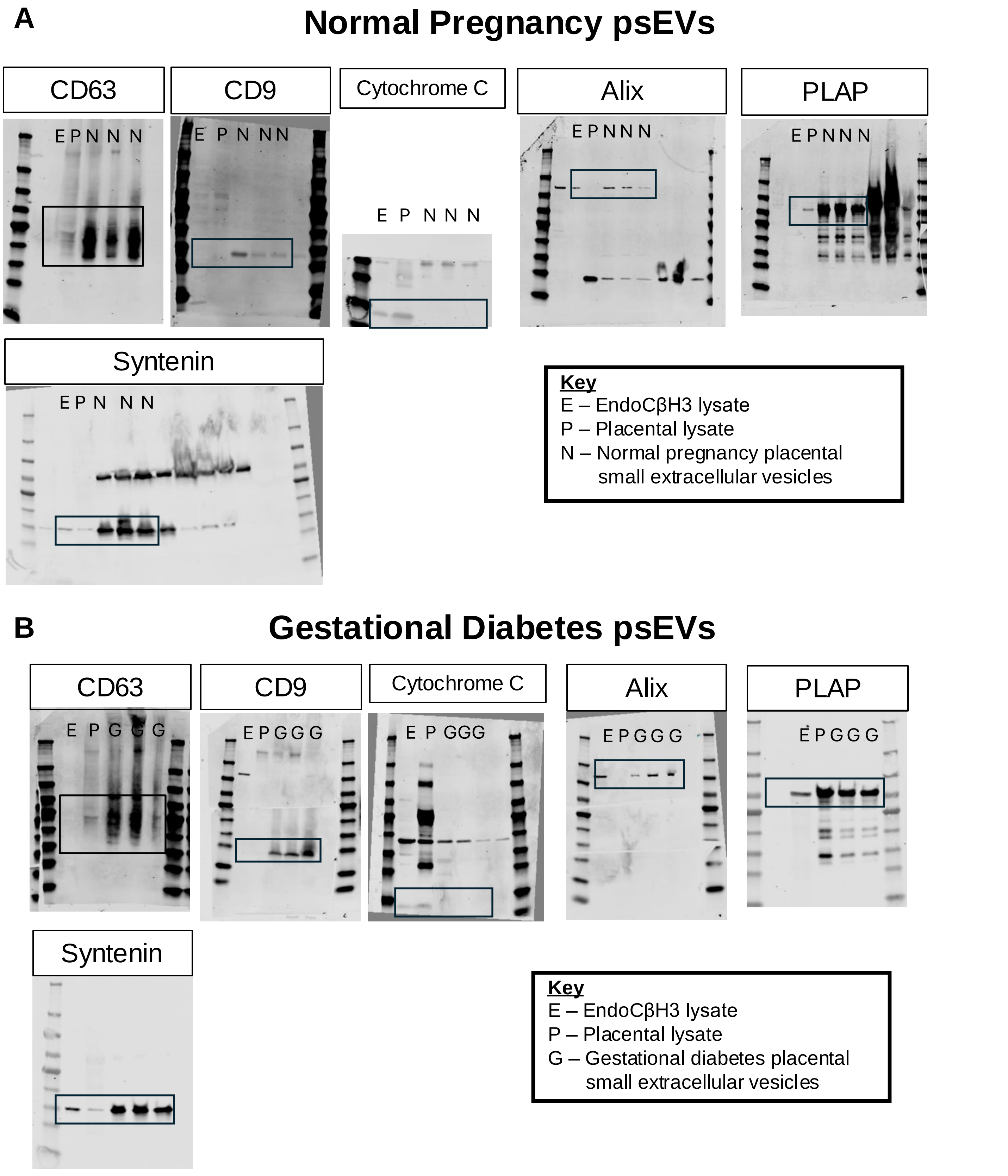

Supplementary Figure S6: Original uncropped immunoblots for Figure 1A and B.

Original unedited immunoblots are shown for **A**. normal pregnancy placental small extracellular vesicles (psEVs) and **B.** gestational diabetes (GDM) psEVs
The proteins of interest for each sub-section are identified by the corresponding headings. The highlighted lanes bound by the box in black indicates the regions that were cropped to create Figure 1A and B in the main text.

A key is provided to match the letters above each lane with the sample loaded into the corresponding well during the experiment.

RBCsEVs

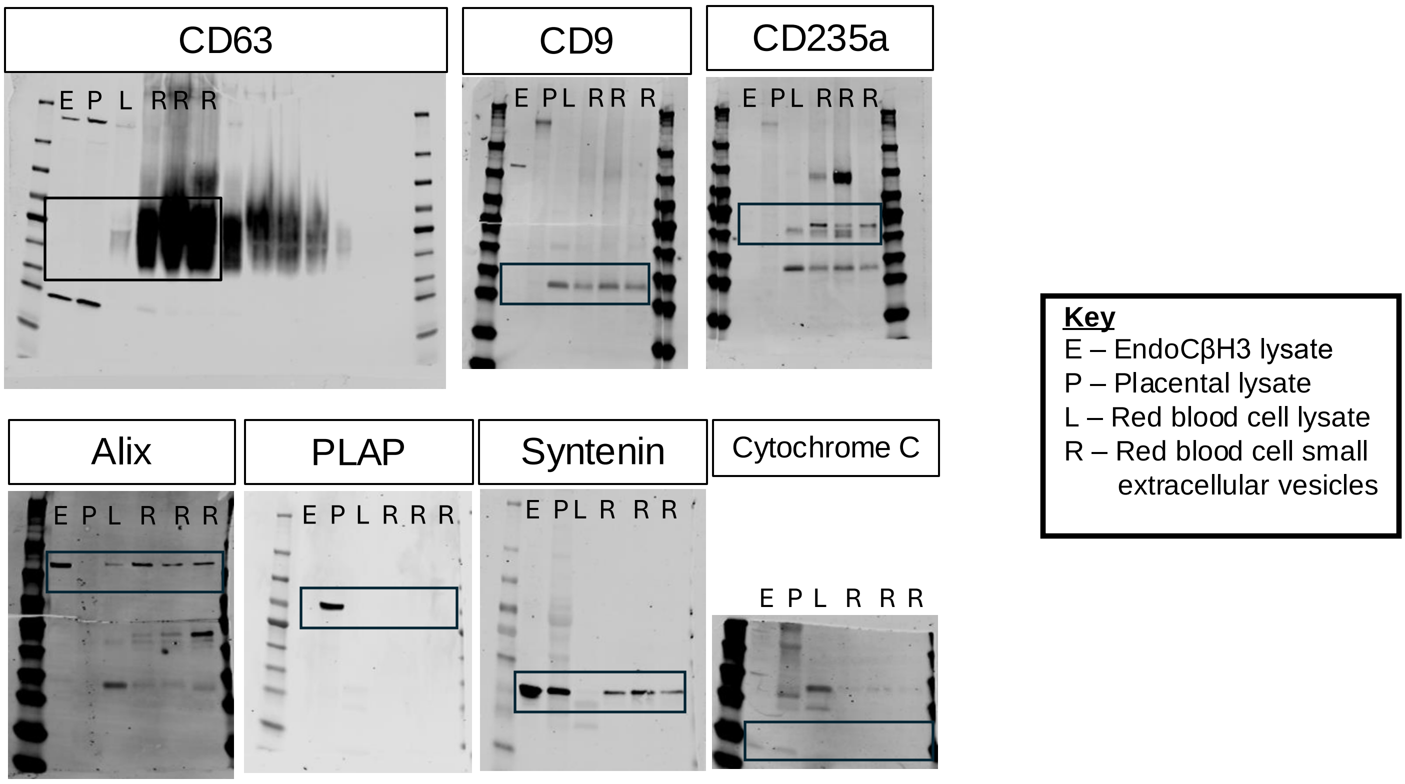

Supplementary Figure S7: Original uncropped immunoblots for Supplementary Figure S1.

Original unedited immunoblots are shown for red blood cell small extracellular vesicles (RBCsEVs).

The proteins of interest for each sub-section are identified by the corresponding headings. The highlighted lanes bound by the box in black indicates the regions that were cropped to create Supplementary Figure S1.

A key is provided to match the letters above each lane with the sample loaded into the corresponding well during the experiment.

KAPCA Recombinant Protein

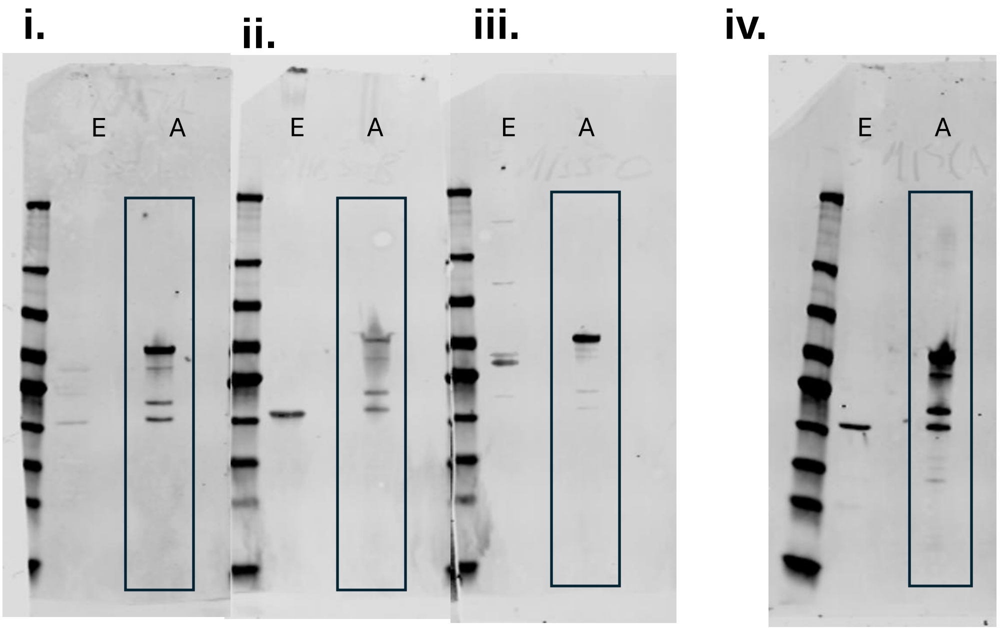

Key
E – EndoCβH3 lysate
A – Recombinant KAPCA protein

Supplementary Figure S8: Original uncropped immunoblots for Figure 7D.

Original unedited immunoblots are shown for Recombinant KAPCA protein (H00005566-P01) using anti-KAPCG antibodies: i. sc-514087 (Santa Cruz Biotechnology USA), ii. ab108385 (Abcam UK), iii. abx301838 (Abbexa UK), iv. Anti-KAPCG antibody (67491-1-IG, Proteintech USA).

The highlighted lanes bound by the box in black indicates the regions that were cropped to create Figure 7D in the main text.

A key is provided to match the letters above each lane with the sample loaded into the corresponding well during the experiment.

Table S1: Antibodies and dyes used.

Antibody/Dye	Concentration	Manufacturer	Catalog Number	Species	Clonality	Dilution
**Immunoblotting**						
Primary antibodies						
Alix 3A9	200µg/ml	Santa Cruz Biotechnology	sc-53538	Mouse	Monoclonal	1 in 200
CD63	200µg/ml	Santa Cruz Biotechnology	sc-5275	Mouse	Monoclonal	1 in 1000
CD9	100µg/ml	Santa Cruz Biotechnology	sc-59140	Mouse	Monoclonal	1 in 200
Glycophorin A (CD235a)	200µg/ml	Santa Cruz Biotechnology	sc-53905	Mouse	Monoclonal	1 in 200
Placental alkaline phosphatase (PLAP)	0.1mg/ml	Abcam	Ab 243731	Rabbit	Polyclonal	1 in 1000
Syntenin	0.54mg/ml	Abcam	Ab 133267	Rabbit	Monoclonal	1 in 1000
Cytochrome c	100µg/ml	Cell Signalling Technology	11940	Rabbit	Monoclonal	1 in 1000
PRKACG (KAPCG)	0.2mg/ml	Santa Cruz Biotechnology	sc-514087	Mouse	Monoclonal	1 in 250
PRKACG (KAPCG)	0.606mg/ml	Abcam	ab108385	Rabbit	Polyclonal	1 in 1000
PRKACG (KAPCG)	4mg/ml	Abbexa	abx301838	Rabbit	Monoclonal	1 in 1000
PRKACA (KAPCA)	1mg/ml	Proteintech	67491-1-IG	Mouse	Monoclonal	1 in 1000
Secondary antibodies						
IRDye 680 anti-Mouse IgG	10mg/ml	LI-COR Biosciences	926-68072	Donkey	-	1in 10000
IRDye 800 anti-Rabbit IgG	10mg/ml	LI-COR Biosciences	926-32213	Donkey	-	1in 10000
**Immunocytochemistry**						
Fluorescent Dyes					-	
PKH 26	1mM	Sigma	PKH26GL-1KT	-	-	
SytoRNA Select	5mM	Thermo Fisher Scientific	S32703	-	-	
WGA 488	1mg/ml	Thermo Fisher Scientific	W11261	-	-	1 in 200
WGA 555	1mg/ml	Thermo Fisher Scientific	W32464	-	-	1 in 200
Hoechst 33342	10mg/ml	Life Technologies	H3570	-	-	1 in 2000
DAPI	1mg/ml	Thermo Fisher Scientific	62248	-	-	1 in 1000
Live Cell Fluorescent Dyes						
MemGlowTM 488	20uM	Cytoskeleton, Inc	MG01-02	-	-	1 in 200
SiR-DNA 647	1mM	Spirochrome	CY-SC007	-	-	1 in 1000
Primary antibodies						
PLAP	0.1mg/ml	Abcam	Ab 243731	Rabbit	Polyclonal	1 in 250
Secondary antibodies						
Anti-Mouse IgG Alexa 488	2mg/ml	Thermo Fisher Scientific	A32723	Goat	Polyclonal	1 in 400
Anti-Rabbit IgG Alexa 488	2mg/ml	Thermo Fisher Scientific	A32731	Goat	Polyclonal	1 in 400
Anti-Rabbit IgG Alexa 555	2mg/ml	Thermo Fisher Scientific	A32732	Goat	Polyclonal	1 in 400

Table S2: Quantitative polymerase chain reaction assays used for RNA detection

Assay type	Manufacturer	Target	Identifier
TaqMan™ MicroRNA Assay	Applied Biosystems	miR 517a	002402
		miR 517c	001153
		miR 518b	001156
		miRN 519a	002415
		U6	001973
TaqMan™ gene expression assay	Applied Biosystems	*INS*	Hs00355773_m1
		TBP	Hs00427620_m1
